# Quercetin as a Multifunctional Flavonol: Molecular Insights and Therapeutic Applications

**DOI:** 10.3390/molecules31162914

**Published:** 2026-08-20

**Authors:** Florina Grumăzescu (Bonifate), Andra-Monica Anghel (Ştefan), Anca Lupu, Elena Enachi, Elena-Lăcrămioara Lisă, Camelia Diaconu

**Affiliations:** Research Centre in the Medical-Pharmaceutical Field, Faculty of Medicine and Pharmacy, “Dunărea de Jos” University, 800008 Galati, Romania; florina.bonifate@ugal.ro (F.G.); andrastefanmonica@gmail.com (A.-M.A.); anca.ravoiu@ugal.ro (A.L.); elena.lisa@ugal.ro (E.-L.L.); camelia.diaconu@ugal.ro (C.D.)

**Keywords:** quercetin, bioavailability, gut microbiota, pharmacological activities, nutraceuticals

## Abstract

Quercetin is one of the most abundant plant flavonoids and an intensively studied bioactive compound belonging to the class of functional heterocyclic compounds with outstanding biological activities, due to its numerous biological, pharmacological and therapeutic effects. This paper presents the main information on the chemical structure, dietary sources, absorption, metabolism and bioavailability of quercetin, highlighting the role of the intestinal microbiota in the formation of metabolites responsible for an important part of its effects. The limitations related to stability and bioavailability are analyzed as well as modern formulation strategies and advanced delivery systems aimed at improving the efficacy of the compound. The major biological activities of quercetin are also summarized, including antioxidant, anti-inflammatory, immunomodulatory, antiviral, cardioprotective, neuroprotective, metabolic and anticancer effects. Unlike previous reviews, which have generally addressed these topics separately, the present work integrates the gut-microbiota-mediated metabolism, cellular senescence, advanced delivery systems, combined therapies, and safety and regulatory considerations within a unified translational framework. Overall, this review emphasizes the need to relate the multifunctional biological potential of quercetin to its pharmacokinetic limitations, formulation performance and clinically achievable exposure while highlighting the need for standardized formulations and further clinical studies to validate its therapeutic benefits.

## 1. Introduction

Quercetin is one of the most abundant flavonoids in the plant kingdom, occurring naturally in many fruits, vegetables and beverages (e.g., tea, onions, apples and berries). Chemically, it is characterized by a complex polyphenolic structure, with multiple hydroxyl groups that contribute to its diverse biological activities [1,2]. Scientific interest in quercetin has expanded considerably over the past decades owing to its broad spectrum of biological activities and its potential applications in both functional nutrition and pharmacology [3].

Biologically, quercetin exhibits a broad spectrum of pharmacological activities, including antioxidant, anti-inflammatory, antiviral, anticancer and cardioprotective effects. These effects are largely mediated by its ability to neutralize reactive oxygen species (ROS), modulate cell signaling pathways and influence gene expression involved in inflammatory and proliferative processes [1,4]. Emerging evidence indicates that quercetin also regulates cellular mechanisms such as apoptosis, cell cycle and energy metabolism, which positions it as a promising candidate in the prevention and therapy of chronic diseases, including cancer, diabetes and cardiovascular diseases [5,6].

Quercetin occupies a unique position at the interface between nutrition and pharmacology, functioning both as a dietary flavonoid and as a bioactive molecule with therapeutic potential [3]. However, despite its favorable biological profile, the clinical applicability of quercetin is limited by its low oral bioavailability, determined by its low aqueous solubility, limited intestinal absorption, and rapid metabolism after oral administration [7,8]. In addition, rapid systemic clearance and extensive transformation into conjugated metabolites reduce the concentration of free quercetin available at the tissue level [9]. Accordingly, recent research has focused on the development of advanced delivery systems, such as nanoemulsions, nanosuspensions, self-nanoemulsifying systems and polymeric nanoparticles, aimed at improving the stability, absorption and biological efficacy of this compound [10].

More recently, the scientific literature has increasingly highlighted the role of quercetin as a multifunctional molecule, with potential applications in personalized medicine and combination therapies. Accumulating evidence suggests that quercetin can act synergistically with different bioactive substances or pharmacological agents, contributing to enhanced therapeutic efficacy while modulating oxidative and inflammatory stress associated with some chronic pathologies [11,12]. In addition, its immunomodulatory and antiviral properties have been investigated, including in the context of emerging viral infections, where quercetin’s ability to modulate the inflammatory response, oxidative stress, and certain mechanisms involved in viral replication was observed [13].

**Figure 1:**
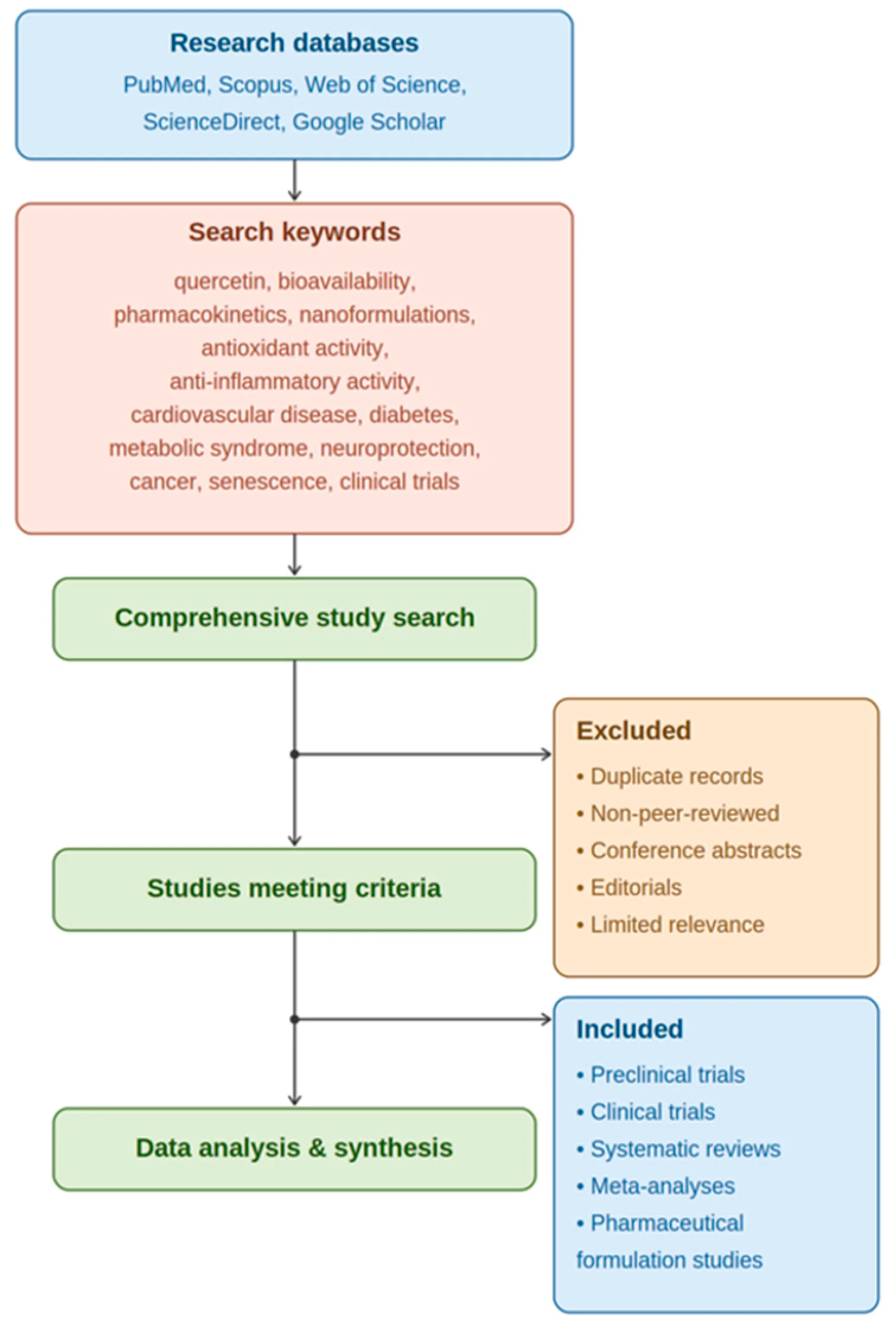
Literature search strategy. Created by the authors based on all the data reported in this manuscript.

Current research increasingly recognizes quercetin as a pleiotropic bioactive molecule acting across interconnected molecular, metabolic and immunological networks. Beyond its classical antioxidant functions, growing attention has been directed toward its interactions with the gut microbiota, regulation of cellular senescence, modulation of neuroinflammatory and immunometabolic pathways, antiviral activity and potential use in combination therapies. At the same time, advances in formulation technologies, nanomedicine and personalized nutrition have highlighted the importance of optimizing quercetin bioavailability and tissue delivery in order to improve its translational potential. Collectively, these advances have broadened the scope of quercetin research from classical antioxidant biology toward systems medicine and precision nutrition. With regard to the literature search strategy (Figure 1), this manuscript focused on a comprehensive search using the PubMed, Web of Science, Google Scholar, and ScienceDirect databases for articles published between 2019 and 2026. Articles were screened based on their title, abstract, and full text. The inclusion criteria targeted peer-reviewed articles, systematic reviews, meta-analyses, clinical investigations, preclinical studies, and pharmaceutical formulation studies directly related to quercetin. Any duplicate records, non-peer-reviewed publications, conference abstracts, editorials, and studies with limited relevance to the review objectives were excluded.

Despite this rapidly expanding literature, the available knowledge remains fragmented. The existing reviews have predominantly provided broad summaries of the pharmacological and clinical potential of quercetin [2,3], whereas others have focused more on its bioavailability limitations and formulation-based approaches for improving its systemic exposure [8,9]. Additional publications have examined selected biological effects or therapeutic applications without consistently relating them to pharmacokinetic behavior, metabolite exposure and formulation performance [10,11,12,13]. The relationships among the physicochemical properties of quercetin; its absorption, distribution, metabolism, excretion and toxicity (ADMET); and its microbiota-dependent biotransformation, environmental stability, formulation-dependent bioavailability and biological activity have not yet been sufficiently integrated. This fragmentation is particularly relevant because biological effects observed at high experimental concentrations cannot be interpreted independently of its pharmacokinetic exposure, while the reported advantages of quercetin nanocarriers require consideration alongside their colloidal stability, formulation-specific limitations, manufacturing reproducibility, shelf life and scalability. Therefore, the present review addresses these gaps by developing an integrated translational framework that links quercetin chemistry and dietary exposure with its complete ADMET profile, gut microbiota-mediated metabolism, environmental stability, formulation-dependent bioavailability and biological activity. Also, this work critically examines how these interconnected factors influence the successful translation of experimental findings into reproducible clinical and nutraceutical outcomes. A particular emphasis is placed on the comparative physicochemical performance and limitations of quercetin-loaded nanocarriers, the barriers to the industrial translation, and emerging areas including cellular senescence, combination therapies, microbiota-dependent variability, safety, and regulatory considerations. By integrating these interconnected directions within a comprehensive translational framework, this review identifies critical gaps in the current knowledge and provides a basis for understanding the pharmacokinetic, formulation, and translational factors that influence the successful application of quercetin in clinical and nutraceutical practice.

## 2. Molecular Determinants of Quercetin Absorption, Metabolism and Bioavailability

### 2.1. Dietary Sources and Nutritional Exposure

Quercetin is one of the most abundant flavonoids in the human diet, being widely distributed in plant foods, especially fruits, vegetables, cereals and beverages such as tea and red wine. It is predominantly found in the form of glycosides (e.g., quercetin-3-O-glucoside or rutin), while its aglycone form is rarely found in natural foods. This chemical distribution significantly influences intestinal absorption and bioavailability [3,7].

**Figure 2:**
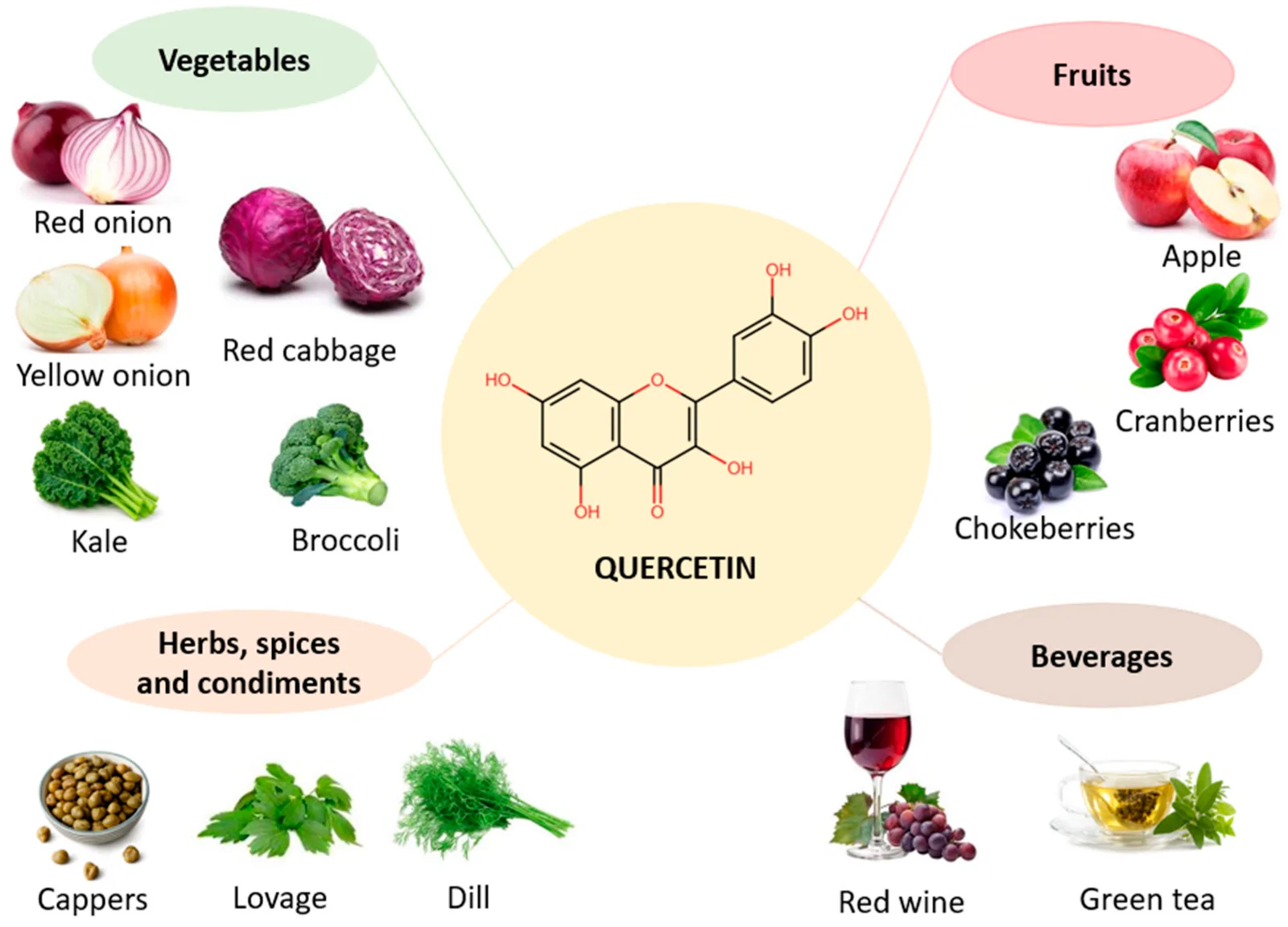
Major dietary sources of quercetin. Created by the authors based on data reported in [4,7].

The main dietary sources of quercetin include onions (especially red and yellow varieties), apples (particularly in the peel), berries, capers, cruciferous vegetables, herbs, spices and beverages such as tea and red wine [14,15]. Among vegetables, onions represent one of the richest dietary sources, with concentrations from approximately 11.7 to 101 mg/100 g FW in red onions and around 20.3 mg/100 g FW in yellow onions, while other important sources include red cabbage (38.6–41.4 mg/100 g FW), kale (22.6 mg/100 g FW) and broccoli (0.03–10.85 mg/100 g FW). Among fruits, cranberries contain approximately 8.3–25 mg/100 g FW and chokeberries around 8.9 mg/100 g FW, whereas apples generally contain lower concentrations (approximately 4.0 mg/100 g FW) despite being widely consumed [4,7,16,17,18]. High quercetin concentrations have also been reported in condiments and aromatic plants, with capers representing the richest commonly consumed dietary source (approximately 332 mg/100 g FW), followed by lovage (170 mg/100 g FW) and dill (40.3–110 mg/100 g FW). In comparison, beverages generally provide lower amounts, with green tea and red wine containing approximately 2.2 and 1.5 mg/100 g FW. Nevertheless, considerable variability exists depending on cultivar, environmental conditions, processing practices, consumption frequency and the predominant occurrence of quercetin as glycosides, all of which influence dietary exposure [4,18]. Some major dietary sources of quercetin are illustrated in Figure 2.

In addition to the total concentration present in food, the nutritional relevance of quercetin is also influenced by the molecular form in which it is consumed. In the plant matrix, quercetin glycosides exhibit different absorption properties compared to the aglycone, and the type of carbohydrate residue can modify both the rate of intestinal absorption and subsequent metabolism. Thus, foods with a comparable content of quercetin can generate different biological responses depending on the glycoside profile and the composition of the food as a whole. In addition, the interaction with dietary fiber, proteins and lipids can influence the release and accessibility of the compound in the gastrointestinal tract [19,20].

The distribution of quercetin in foods is influenced by numerous factors, including plant genotype, growing conditions, light exposure, maturity at harvest, and processing and storage methods. In many cases, the highest concentrations are located in peripheral tissues of the plant, such as the skin of fruits or the outer layers of bulbs and leaves. Also, heat treatments can reduce the total content through oxidative degradation or loss in the preparation environment, but certain technological processes can favor the release of bound compounds and increase their biological accessibility [21].

From a nutritional point of view, the daily intake of quercetin varies considerably between populations and dietary patterns, being influenced both by the frequency of consumption of plant products and by the diversity of food sources. Available estimates indicate general values ranging from approximately 5 to 100 mg/day, but diets rich in fruits, vegetables and plant-based beverages may provide higher amounts. In this context, quercetin is considered a relevant nutraceutical compound and one of the markers of increased polyphenol intake, being frequently associated with the beneficial effects attributed to Mediterranean-type or plant-based diets [22,23].

The isolation and quantification of quercetin from plant matrices and food products are essential steps for the assessment of the real content and for the correct interpretation of the biological effects attributed to this flavonol. The complexity of these processes is determined both by the natural distribution of quercetin in a predominantly glycosylated form and by the heterogeneous composition of plant matrices, which contain numerous flavonoids and structurally related phenolic compounds with similar chromatographic and spectroscopic behavior. Consequently, the choice of extraction method and analytical technique directly influences the recovery efficiency, separation selectivity and quantification accuracy [2,3].

An important aspect in determining the total quercetin content is the chemical form analyzed. Since quercetin naturally occurs predominantly as glycosylated derivatives, including quercetin glucosides and rutinosides, many protocols include an acidic or enzymatic hydrolysis step to convert glycosylated derivatives to aglycone. This approach facilitates the comparability of results and allows for more accurate estimation of the total quercetin content between different food or botanical sources [20].

### 2.2. Structural and Physicochemical Properties

**Figure 3:**
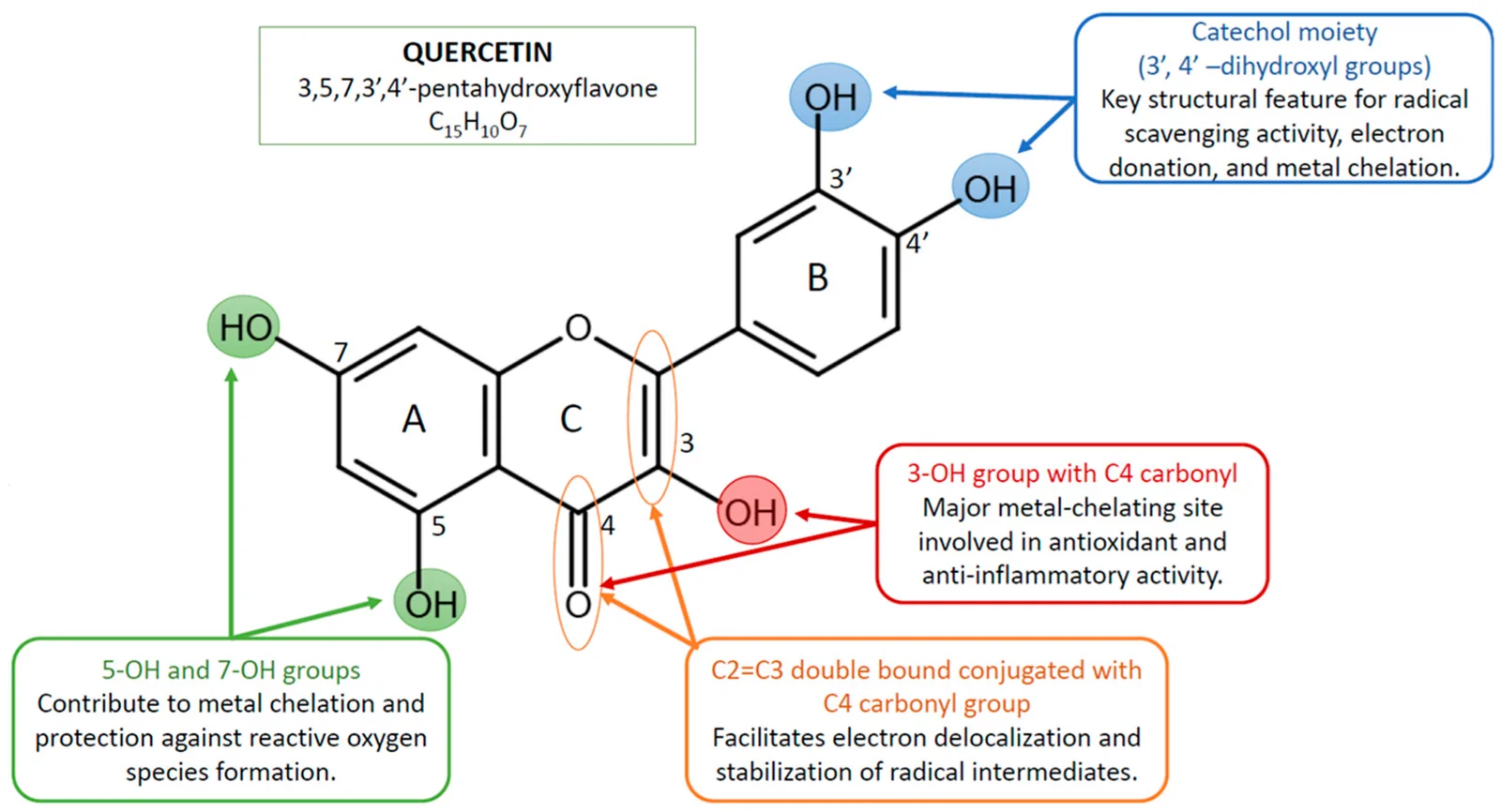
Chemical structure of quercetin and major structure–activity relationship (SAR) features underlying its biological activity. Created by the authors based on data reported in [25,26].

Quercetin (Figure 3), systematically called 3,5,7,3′,4′-pentahydroxyflavone, is a polyphenolic flavonol belonging to the flavonoid family, with the molecular formula C_15_H_10_O_7_ and a molecular mass of approximately 302.24 g/mol. Structurally, the molecule presents the typical C6–C3–C6 flavonoid backbone, consisting of two aromatic rings, A and B, connected by an oxygenated heterocycle, ring C. The presence of the five hydroxyl groups, located at positions 3, 5, 7, 3′ and 4′, gives quercetin a pronounced polyphenolic character and largely explains its ability to participate in redox reactions, form hydrogen bonds and chelate transition metal ions. Owing to these structural features, quercetin exhibits remarkable chemical reactivity and has been extensively investigated for its antioxidant and biologically active properties [15,24].

Structurally, the biological activity of quercetin is directly influenced by the organization of the functional groups in the flavonol nucleus. The catechol system present on the B ring, the C2=C3 double bond conjugated to the carbonyl function at C4, and the hydroxyl group at the C3 position contribute to electron stabilization and increase the antioxidant capacity of the molecule. This extensive conjugation facilitates electron transfer and the neutralization of reactive oxygen species, explaining the ability of quercetin to act as a radical scavenger and chelating agent for transition metal ions [16].

At the same time, the redox properties of quercetin are influenced by environmental conditions, including pH, the presence of metal ions and the chemical form of the compound, free or glycosylated. In addition, the hydroxyl groups involved in antioxidant activity can participate in complex intermolecular interactions, such as the formation of hydrogen bonds or metal complexes, aspects relevant to the stability, solubility and pharmacokinetic behavior of the molecule [27].

In its pure state, quercetin is described as a yellow crystalline powder, poorly soluble in water but with higher solubility in organic solvents such as ethanol, methanol, dimethyl sulfoxide or alkaline solutions. Quantitative physicochemical parameters further illustrate the limitations associated with quercetin bioavailability. The aglycone exhibits very low aqueous solubility under neutral conditions, typically reported in the low microgram-per-milliliter range, while its lipophilicity is reflected by a logP value of approximately 1.5–2.0. In addition, quercetin possesses multiple ionizable hydroxyl groups with pKa values ranging from approximately 6 to 11, depending on the hydroxyl position, which contributes to pH-dependent changes in solubility, membrane permeability, and oxidative stability [3,7,8]. This low aqueous solubility represents one of the main limitations of its biological and pharmaceutical use. Recent studies show that the low bioavailability of quercetin is influenced by several factors, including relative hydrophobicity, limited intestinal permeability, instability in the gastrointestinal environment and extensive metabolism after oral administration [8].

Also, natural glycosylated forms, such as isoquercitrin or rutin, may exhibit different absorption and distribution profiles compared with the aglycone depending on the structure of the carbohydrate residue and the intestinal transport mechanisms involved. The nature of the attached sugar moiety influences intestinal absorption, enzymatic hydrolysis, and systemic bioavailability, resulting in differences in biological activity among individual quercetin derivatives [7]. Thus, the chemical structure of quercetin influences not only its antioxidant properties but also its pharmacokinetic behavior and biological efficacy in vivo.

The acid–base properties of quercetin derive from the phenolic character of the hydroxyl groups. At physiological pH, the molecule can exist partially in ionized forms, which influences solubility, oxidative stability and interaction with plasma proteins or biological membranes. However, the increase in the degree of ionization in alkaline medium is associated with increased susceptibility to oxidation and degradation, an aspect highlighted by recent studies on the stability of quercetin under different pH and temperature conditions [28]. For this reason, careful control of pH is an important element both in in vitro experiments and in the development of pharmaceutical and nutraceutical formulations. This duality—enhanced solubility under alkaline conditions but potentially reduced stability—has supported the development of modern delivery systems designed to protect the compound and optimize bioavailability. In recent years, nanoparticles, liposomes, micelles, and other nanoformulations have been extensively investigated to increase the stability and biological efficacy of quercetin [9].

Another important aspect of the physicochemical profile of quercetin is its ability to form intermolecular and supramolecular complexes, a property exploited in the development of modern formulation systems. Interactions with phospholipids, cyclodextrins, natural polymers or nanoparticles can significantly modify the solubility, stability and pharmacokinetic behavior of the compound, contributing to increased bioavailability and protection against oxidative degradation. In addition, the polyphenolic character of quercetin favors interactions with proteins and biological membranes, an aspect relevant for tissue distribution and biological activity [8,9].

When considered collectively, the physicochemical profile of quercetin is characterized by a close relationship between structure, reactivity, and bioavailability. The five hydroxyl groups and the conjugated π system explain the antioxidant potential and the capacity for molecular interaction, but the same architecture contributes to low aqueous solubility and environment-dependent stability.

### 2.3. ADMET Profile and Microbiota-Dependent Bioavailability of Quercetin

The ADMET profile of quercetin is a major determinant of its therapeutic and nutraceutical applicability. Despite the biological effects demonstrated in numerous experimental studies, only a limited fraction of orally administered quercetin reaches the systemic circulation. Its pharmacokinetic behavior is influenced by aqueous solubility, chemical form, intestinal permeability, food matrix, formulation technology, presystemic metabolism and interindividual variability [29,30].

Absorption: Quercetin is absorbed predominantly in the small intestine, although the extent and rate of absorption depend strongly on its molecular form. In foods, quercetin occurs mainly as glycosides, which require enzymatic hydrolysis before or during intestinal uptake. Glucose-containing glycosides may be hydrolyzed at the intestinal brush border or transported into enterocytes, whereas rutin and other glycosides that are less efficiently processed in the small intestine may reach the colon and undergo microbial degradation. The aglycone is limited by its poor aqueous solubility, and absorption may therefore vary considerably according to the administered formulation [29,30].

Distribution: Following intestinal absorption, quercetin is present in the circulation predominantly as glucuronidated, sulfated and methylated conjugates rather than as free aglycone. These metabolites interact with plasma proteins and are distributed to metabolically active tissues, particularly the liver, kidneys and intestinal tissues. However, human data regarding tissue-specific concentrations remain limited, and distribution profiles are influenced by the chemical form, administered dose, formulation and metabolic status of the individual. Consequently, plasma concentrations of total quercetin metabolites do not necessarily reflect the concentrations of biologically active forms available within specific tissues [3,14].

Metabolism: Quercetin undergoes extensive phase II metabolism in enterocytes and the liver, primarily through glucuronidation, sulfation and methylation. Consequently, the parent aglycone is detected only at very low concentrations in plasma, whereas conjugated derivatives represent the predominant circulating forms [31,32]. These metabolic transformations influence tissue distribution and biological activity, indicating that the in vivo effects of quercetin result from the combined activity of the parent compound and its metabolites rather than exclusively from the unconjugated aglycone [32,33].

The fraction that is not absorbed in the small intestine reaches the colon, where it undergoes microbial deglycosylation, ring fission and further conversion into lower-molecular-weight phenolic compounds, including phenylpropionic, phenylacetic and benzoic acid derivatives. These microbial metabolites may subsequently be absorbed through the colonic epithelium and undergo additional hepatic conjugation. Differences in gut microbiota composition may therefore contribute to the substantial interindividual variability observed in quercetin metabolism and biological responsiveness. Increasing evidence suggests that these microbiota-derived metabolites retain biological activity and may contribute to the antioxidant, anti-inflammatory and metabolic effects traditionally attributed to quercetin. Consequently, its in vivo activity should be regarded as the integrated outcome of the parent compound, phase II conjugates and gut-microbiota-derived phenolic metabolites rather than the effect of the aglycone alone [34,35,36].

Excretion: Quercetin-derived metabolites are eliminated primarily through the kidneys after conversion into more water-soluble glucuronidated and sulfated conjugates. Biliary secretion may also contribute to their elimination and may permit partial enterohepatic recirculation, thereby prolonging the systemic presence of selected metabolites. Gut-microbiota-derived phenolic metabolites are similarly eliminated mainly through urine after intestinal absorption and hepatic conjugation. The relative contribution of renal and biliary elimination depends on the molecular form, administered dose, formulation and metabolic pathway involved [3,32].

Toxicity: Quercetin generally shows a favorable safety profile at dietary exposure levels and within the doses and intervention periods evaluated in short-term human studies. Nevertheless, safety cannot be inferred exclusively from its natural occurrence in foods, particularly when concentrated supplements or bioavailability-enhanced formulations are used. At high exposure levels, potential prooxidant effects, interactions with drug-metabolizing enzymes and transporters, and possible hepatic or renal effects require consideration. Evidence regarding prolonged supplementation, vulnerable populations and formulations producing substantially increased systemic exposure remains limited [3,17]. Therefore, the toxicity profile of quercetin should be interpreted in relation to dose, duration, chemical form, formulation-dependent bioavailability, concomitant medication and individual susceptibility.

**Figure 4:**
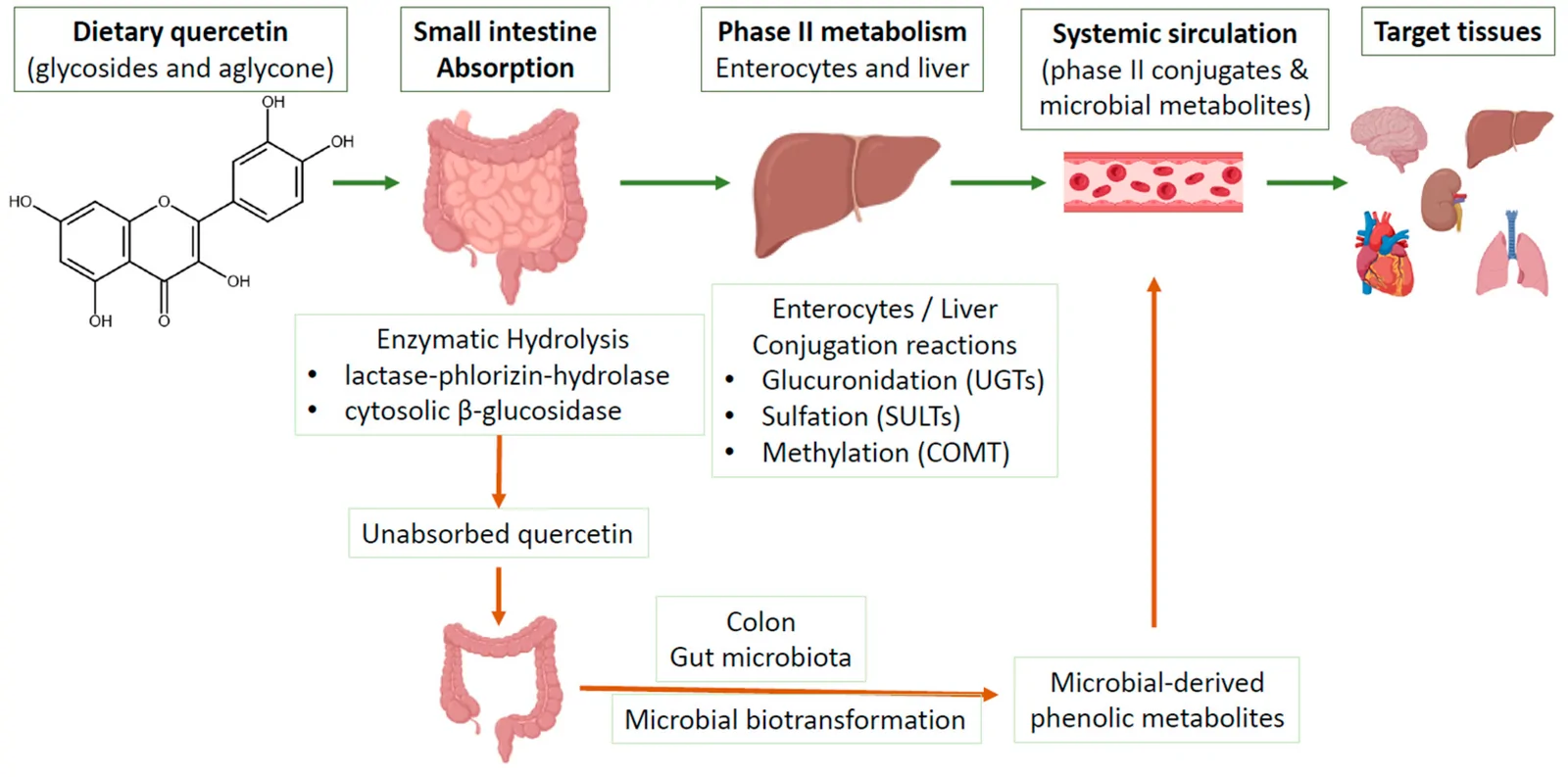
Bioavailability and metabolism of quercetin. Created by the authors based on data reported in [19,36].

The ADMET profile of quercetin is strongly influenced by its physicochemical properties, administered chemical form, food or pharmaceutical matrix, gut microbiota composition and formulation technology. These factors determine not only the extent of intestinal absorption but also metabolite formation, tissue exposure, elimination and safety. Therefore, the biological effects of quercetin should be interpreted in relation to the systemic exposure achieved in vivo rather than solely to the administered dose. The major processes governing quercetin absorption, distribution, metabolism and elimination are summarized in Figure 4.

## 3. Gut-Microbiota-Mediated Metabolism of Quercetin and Its Implications for the Gut–Organ Axes

Interactions between quercetin and the gut microbiota are increasingly recognized as important determinants of quercetin’s biological activity because intestinal microorganisms directly influence flavonoid biotransformation, metabolite generation and systemic exposure. Due to incomplete absorption in the small intestine and extensive phase II metabolism, a substantial fraction of ingested quercetin reaches the colon, where it undergoes microbial conversion into lower-molecular-weight phenolic metabolites with biological properties that may differ from those of the parent compound. Microbial catabolism involves deglycosylation, dehydroxylation, ring fission and additional biotransformation reactions that generate phenolic derivatives capable of entering systemic circulation and contributing to host physiological responses associated with quercetin intake [31,37]. These observations indicate that the biological effects observed in vivo cannot be interpreted solely through intact quercetin, since microbially derived metabolites may substantially influence tissue exposure, downstream signaling responses and metabolic fate. Moreover, interindividual differences in microbiota composition may alter metabolite generation efficiency and microbial processing pathways, contributing to variability in biological responsiveness between individuals and across experimental or clinical studies [35,37].

Quercetin may also modulate gut microbiota composition and intestinal barrier function, supporting a bidirectional interaction between the compound and the host–microbial ecosystem. Experimental studies indicate that quercetin can improve microbial diversity, reduce dysbiosis-associated alterations and favor bacterial groups involved in intestinal homeostasis, effects that may contribute to reduced mucosal inflammation and improved metabolic regulation [34,36]. In parallel, quercetin has been associated with restoration of tight-junction integrity, reduced intestinal permeability and increased production of metabolites involved in epithelial protection, including short-chain fatty acids and tryptophan-derived compounds [35,36]. These effects are particularly relevant because disruption of the intestinal barrier facilitates translocation of microbial products, promotes low-grade inflammation and contributes to systemic metabolic and immune dysregulation. Therefore, the capacity of quercetin to support epithelial barrier integrity may represent a key mechanism linking its local intestinal actions with systemic biological effects.

The metabolic consequences of quercetin–microbiota interactions extend beyond the intestinal lumen. Microbially derived phenolic metabolites and short-chain fatty acids may participate in the regulation of host inflammatory and metabolic pathways by modulating oxidative stress, immune cell activation and epithelial–immune communication [18,38]. In this context, quercetin may act both as a substrate for microbial metabolism and as a modulator of microbial ecology. This bidirectional relationship is important for understanding why the same quercetin dose may produce different biological responses depending on gut microbial composition, dietary background, formulation and host metabolic status. Consequently, microbiota-mediated metabolism should be considered an essential component of quercetin pharmacology, particularly when interpreting data from oral administration studies.

The gut–liver axis represents one of the most relevant pathways through which quercetin–microbiota interactions may influence systemic health. Increased intestinal permeability and dysbiosis can facilitate the passage of microbially derived products toward the portal circulation, promoting hepatic inflammation, oxidative stress and metabolic dysfunction. By improving intestinal barrier integrity and modulating microbial metabolites, quercetin may indirectly contribute to reduced hepatic inflammatory burden and improved metabolic homeostasis, mechanisms particularly relevant in obesity, insulin resistance and metabolic-dysfunction-associated steatotic liver disease [15,19]. These observations support the idea that the metabolic effects of quercetin are not limited to direct actions on hepatic or adipose tissues but may also involve upstream regulation of intestinal microbial and barrier-related processes.

In addition to the gut–liver axis, the gut–brain axis has emerged as a potential pathway linking quercetin intake, microbiota modulation and neuroprotective outcomes. Gut microbial metabolites can influence neuroinflammatory signaling, oxidative stress responses and immune–neural communication, thereby contributing to the regulation of neuronal homeostasis. Although the direct clinical relevance of this pathway remains insufficiently established, experimental evidence suggests that quercetin-mediated improvement of gut microbial balance and intestinal barrier function may indirectly support neuroprotective effects by reducing systemic inflammatory signals that contribute to neuroinflammation [18,19]. This perspective is consistent with the broader view that quercetin acts as a multitarget bioactive compound whose effects depend on the integration of intestinal metabolism, microbial ecology and host signaling networks.

Despite increasing interest in this topic, several limitations remain. Most evidence regarding quercetin–microbiota interactions is derived from in vitro fermentation systems, animal models or small-scale experimental studies, while human data remain comparatively limited. In addition, differences in quercetin formulation, dose, dietary background, baseline microbiota composition and analytical methods complicate direct comparison between studies [31,37]. Future research should therefore integrate metagenomic, metabolomic and pharmacokinetic approaches to clarify how microbial biotransformation influences circulating metabolite profiles and biological responses. Such studies are essential for determining whether microbiota-targeted strategies or personalized nutrition approaches could improve the efficacy of quercetin-based interventions.

However, most microbiota-related findings derive from in vitro fermentation or animal studies, and differences in experimental models, quercetin forms, doses and sequencing methodologies limit direct comparison.

## 4. Quercetin Stability Challenges and Modern Delivery Systems

### 4.1. Molecular Basis of Quercetin Instability

The stability of quercetin is a key determinant of its biological activity and practical application in food, nutraceutical and pharmaceutical products [38]. Although quercetin is widely recognized for its potent antioxidant properties and ability to neutralize reactive oxygen species, its polyphenolic structure also renders it susceptible to degradation under various environmental conditions [9,39]. The presence of five hydroxyl groups and an extended conjugated π-electron system promotes electron transfer and redox activity but simultaneously increases susceptibility to oxidation and structural transformations during extraction, processing, storage and formulation [33]. Consequently, quercetin may gradually lose its physicochemical integrity and biological activity unless appropriate stabilization strategies are implemented [3,40,41].

### 4.2. Factors Influencing Quercetin Stability

Among the variables influencing quercetin stability, pH is considered one of the most critical determinants. Experimental studies have consistently shown that quercetin remains comparatively stable under acidic conditions, whereas neutral and especially alkaline environments accelerate oxidative degradation. Progressive deprotonation of phenolic hydroxyl groups increases molecular reactivity, promoting autooxidation, structural rearrangements and the formation of secondary oxidation products, which reduce the concentration of intact quercetin and may alter its antioxidant performance [28,39]. Thermal processing under aqueous conditions may further enhance these degradation reactions through oxidation, hydroxylation and molecular cleavage [42]. Therefore, careful pH control is essential not only during extraction and analytical procedures but also in the design of pharmaceutical and nutraceutical formulations intended to preserve quercetin prior to absorption.

In addition to pH, environmental factors, such as light, oxygen, humidity and temperature, play a major role in determining quercetin stability during processing and storage. Exposure to atmospheric oxygen and ultraviolet radiation promotes photooxidation and the formation of reactive intermediates, whereas high humidity further accelerates degradation processes [43,44]. Thermal treatment also contributes to progressive decomposition through oxidation and structural transformation reactions, although the magnitude of these effects depends on the processing conditions and surrounding matrix. Interestingly, while elevated temperatures may reduce the concentration of intact quercetin, thermal processing of complex food matrices can simultaneously enhance compound release and bioaccessibility, partially compensating for degradation losses [45,46]. Consequently, appropriate control of storage and processing conditions is essential to preserve quercetin stability throughout the product life cycle.

Quercetin’s chemical form also influences its stability profile. Glycosylated derivatives generally exhibit greater resistance to environmental degradation than the free aglycone, particularly in aqueous media. In addition, interactions with proteins, polysaccharides and lipid structures can protect quercetin from oxidation by limiting its direct exposure to oxygen and reducing molecular mobility. Such interactions are particularly relevant in functional foods and nutraceutical formulations, where quercetin is commonly incorporated into complex matrices rather than administered as an isolated compound [27,47].

### 4.3. Advanced Delivery Strategies

To overcome the physicochemical and pharmacokinetic limitations of quercetin, considerable research has focused on the development of advanced delivery systems capable of protecting the compound during storage and gastrointestinal transit while simultaneously improving its solubility, intestinal absorption and systemic availability. Among the most extensively investigated approaches are liposomes, polymeric nanoparticles, lipid-based carriers, nanoemulsions, self-emulsifying drug delivery systems and controlled-release platforms. In addition to enhancing physicochemical stability, these formulations may improve oral bioavailability and contribute to more efficient tissue delivery compared with conventional preparations [48,49]. Recent advances have further expanded the development of smart and multifunctional delivery systems capable of controlling release kinetics, prolonging gastrointestinal residence time and reducing interindividual variability in absorption. Some formulation strategies may also influence the microbial biotransformation, thereby further contributing to an improved intestinal availability of quercetin [34,35]. Although these nanoformulations have demonstrated promising results in experimental models, additional well-designed clinical studies are still required to establish standardized delivery strategies and confirm their long-term therapeutic benefits in humans [48,49,50].

The performance of quercetin-loaded nanocarriers depends not only on particle size and encapsulation efficiency but also on surface charge, size distribution, carrier composition and release behavior. Particle size influences gastrointestinal transport, cellular uptake and physical stability, whereas zeta potential provides an indication of the electrostatic interactions that contribute to colloidal stability. Nevertheless, zeta potential should not be interpreted independently, because steric stabilization, lipid composition and polymer coating may maintain dispersion stability even when the absolute surface charge is relatively low. Consequently, direct comparison between nanocarriers requires consideration of several complementary physicochemical parameters rather than reliance on a single indicator [48,49,50].

Lipid-based platforms, including liposomes, nanoliposomes, nanostructured lipid carriers and nanocochleates, are particularly suitable for quercetin because their hydrophobic domains facilitate incorporation of the poorly water-soluble aglycone. Liposomes and nanoliposomes can protect quercetin against environmental and gastrointestinal degradation and provide sustained release, but their performance is strongly influenced by phospholipid composition, cholesterol content, preparation method and storage conditions. Potential limitations include moderate encapsulation efficiency, phospholipid oxidation, drug leakage, aggregation and the need for controlled storage. Nanostructured lipid carriers generally provide higher encapsulation efficiency and improved colloidal stability, although their characteristics vary according to the lipid and oil composition, and some formulations remain primarily suitable for topical rather than systemic administration. Nanocochleates offer a compact lipid structure with prolonged release potential, but their larger or heterogeneous particle populations and limited clinical validation restrict direct comparison with conventional nanoliposomes [28,51,52,53,54,55].

**Figure 5:**
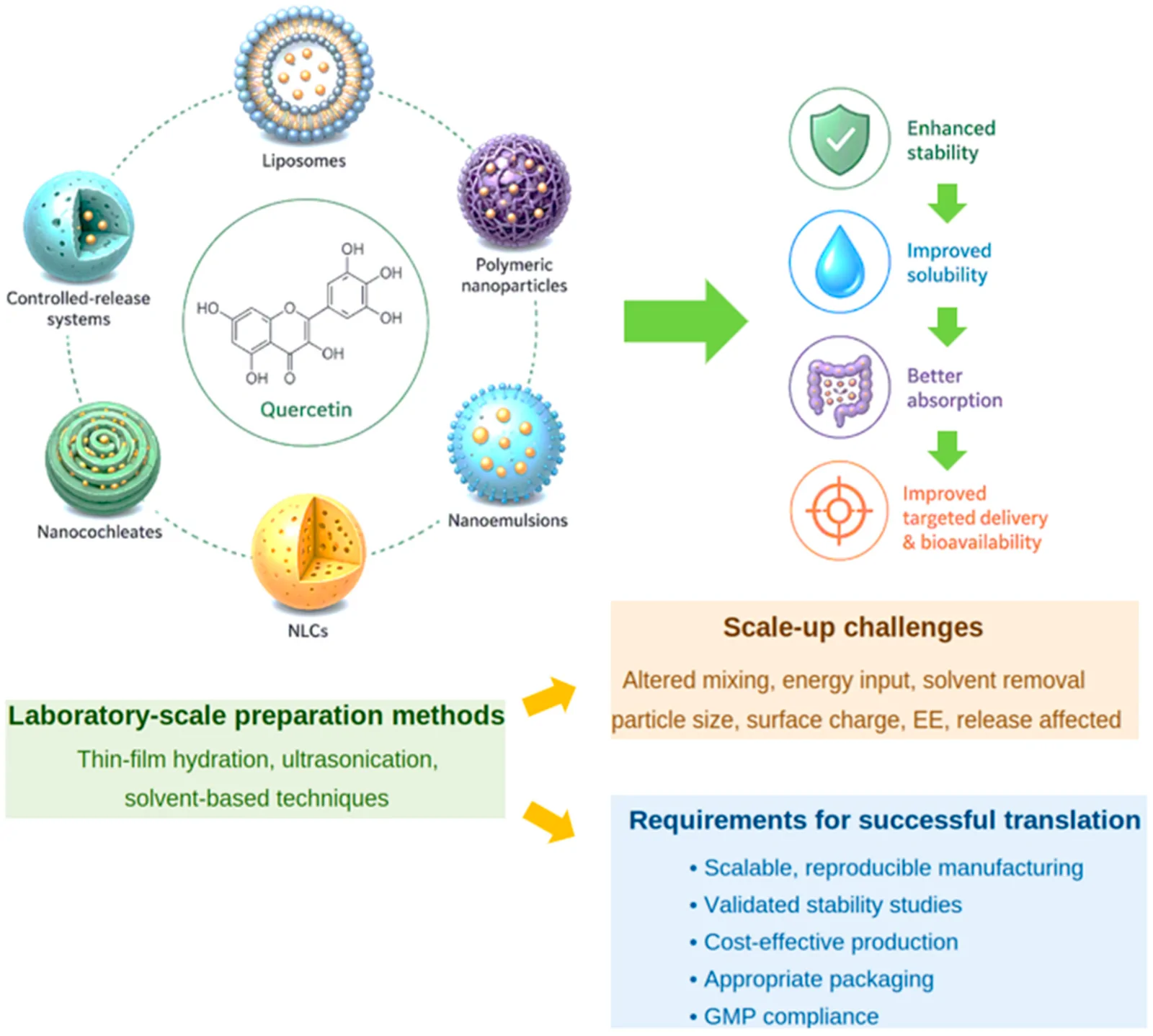
Nanotechnology-based delivery systems for quercetin: benefits and scale-up considerations. Created by the authors based on the data reported in this section.

Polymeric nanoparticles and biopolymer-based systems provide additional opportunities for mucoadhesion, protection during gastrointestinal transit and controlled or site-specific release. Chitosan-containing carriers may enhance interaction with biological membranes because of their positive surface charge, although aggregation, pH-dependent behavior and variability in polymer characteristics may affect reproducibility. Pickering emulsion gels and alginate–chitosan microgels can improve gastrointestinal protection, bioaccessibility or colon-targeted delivery, but their micrometric dimensions distinguish them from conventional nanosystems and may limit systemic absorption [23,34,52]. As shown in Figure 5, various nanotechnology-based delivery systems have been explored to improve the pharmaceutical performance of quercetin, including its stability, solubility, absorption, and targeted bioavailability.

Despite their promising experimental performance, the industrial translation of quercetin-loaded nanocarriers remains challenging. Many preparation methods used at laboratory scale, including thin-film hydration, ultrasonication and solvent-based techniques, are difficult to transfer directly to large-scale manufacturing because changes in mixing conditions, energy input and solvent removal may alter particle size, surface charge, encapsulation efficiency and release behavior. Batch-to-batch reproducibility therefore requires strict control of raw-material characteristics and critical process parameters, together with standardized quality attributes. Shelf-life represents an additional concern, particularly for lipid-based carriers, which may undergo phospholipid oxidation, aggregation, fusion or leakage of encapsulated quercetin during storage.

Polymeric systems may similarly be affected by variations in polymer molecular weight, purity and degradation behavior. Successful translation will consequently require scalable and reproducible manufacturing technologies, validated stability studies under relevant storage conditions, cost-effective production, appropriate packaging and compliance with Good Manufacturing Practice requirements. These considerations should be evaluated together with the reported improvements in stability, bioavailability and therapeutic efficacy, as promising laboratory-scale performance does not necessarily ensure industrial or clinical feasibility [47,48,49,50].

A few of the most representative examples of nanoencapsulation systems developed to improve quercetin stability, encapsulation efficiency and delivery performance are summarized in Table 1, together with their particle size, zeta potential, principal advantages and main limitations.

The encapsulation strategies discussed above address the physicochemical instability of quercetin more convincingly than its pharmacokinetic variability. Until the various delivery systems are directly compared in standardized human clinical studies, not just in isolated preclinical models, claims of superior bioavailability will remain difficult to compare between these studies and hard to translate into clinical practice.

## 5. Clinical Translation of Quercetin and Regulatory Challenges

### 5.1. Translating Experimental Evidence into Clinical Practice

Dietary quercetin and quercetin taken as a supplement should not be considered biologically equivalent. Although both represent the same class of bioactive compound, the context of administration significantly influences bioavailability and physiological response. In the diet, quercetin is consumed in moderate concentrations and is integrated into a complex food matrix together with fiber, carbohydrates, lipids, proteins and numerous other phenolic compounds, whereas supplements frequently provide concentrated and standardized doses designed to achieve higher systemic exposure than a typical dietary intake [56,57]. This distinction is important because the food matrix influences quercetin’s release, intestinal accessibility, metabolism, absorption and tolerability compared with its isolated administration as a supplement [37]. Furthermore, quercetin supplements are available in multiple forms, including aglycone, quercetin dihydrate, glycosides and bioavailability-enhanced formulations, which are not pharmacokinetically equivalent [58]. Recent evidence indicates that systemic exposure is determined by the combined influence of the chemical form, aqueous solubility, gastrointestinal stability, intestinal and hepatic metabolism, formulation technology and interindividual variability associated with the gut microbiota [30]. Consequently, identical nominal doses may result in markedly different systemic exposure depending on both the formulation and the individual receiving it [59].

From a practical perspective, the dietary intake of quercetin is generally preferable as a public health strategy because it is associated with diets rich in fruits and vegetables rather than the isolated administration of a single compound [56]. However, supplementation may be relevant in experimental, clinical and nutraceutical settings where controlled doses are required or when the focus is to overcome the pharmacokinetic limitations of dietary intake [60].

Nevertheless, the interpretation of the reported benefits requires caution. Many effects attributed to quercetin have been observed under experimental conditions or at concentrations that may not reflect those achieved after a routine oral administration in humans [12]. In addition, commercial products may differ substantially in purity, stability, excipients and actual bioavailability, highlighting the need for standardization and independent evaluation [61]. Although the safety profile of quercetin appears favorable in short-term clinical studies [62], current research remains insufficient to draw firm conclusions regarding its long-term administration, particularly at high doses or when bioavailability-enhanced formulations are used [14]. When it comes to pregnant or lactating women, children, individuals with renal or hepatic disease and patients receiving medications with a narrow therapeutic index, severe caution must be exercised, as quercetin may influence the transporters and enzymes involved in xenobiotic metabolism [57]. As such, supplementation should be regarded as a formulation- and dose-dependent nutraceutical intervention, whereas dietary quercetin intake remains part of a broader and multifactorial dietary pattern.

### 5.2. Clinical Evidence and Safety Considerations

Commercial quercetin supplements are currently available in several chemical forms and formulation technologies designed to improve oral performance and product stability. These include quercetin aglycone, quercetin dihydrate, glycosides, phospholipid complexes (phytosomes), liposomal formulations, micellar systems, nanoemulsions, polymeric nanoparticles and cyclodextrin inclusion complexes. The selected formulation influences not only intestinal absorption but also stability, tissue distribution and systemic exposure [58].

Lipid and phospholipid formulations have attracted particular interest because they can facilitate gastrointestinal dispersion and improve the transfer of the compound to the intestinal absorption surface. In parallel, micellar systems and nanostructured formulations have been developed to reduce the limitations imposed by the hydrophobicity of the molecule and to increase oral availability without a proportional increase in the administered dose [59].

Recent pharmacokinetic data suggest that optimized formulations may produce important differences in systemic exposure parameters compared to conventional quercetin, including peak plasma concentration and total availability after administration. These observations support the idea that two products that claim the same amount of quercetin should not automatically be assumed to be biologically equivalent [30].

Beyond improving intestinal absorption, advanced formulations are also designed to preserve quercetin during storage and gastrointestinal transit, thereby limiting chemical degradation before systemic absorption. Consequently, current formulation strategies aim to integrate enhanced stability with improved pharmacokinetic performance and biological efficacy [60].

From a practical perspective and for the correct interpretation of the supplement market, the evaluation of formulations should go beyond the simple analysis of the declared dose. For relevant comparisons between products, it is recommended to include criteria such as chemical form, formulation technology, dose per serving, excipients used, the existence of pharmacokinetic data and the level of quality control [61].

The oral bioavailability of quercetin is limited by its low water solubility, moderate intestinal permeability, and extensive presystemic metabolism in the intestine and liver. As a result, numerous formulation systems have been developed to improve the absorption and systemic exposure of the compound. Among the most effective are phytosomes (quercetin–phospholipid complexes), liposomal systems, and other nanoformulated platforms, which can increase quercetin’s apparent solubility, facilitate the crossing of biological membranes, and may promote lymphatic absorption. In contrast, conventional crystalline quercetin has a low bioavailability and is frequently used as a reference form for evaluating the performance of new delivery systems. Some representative commercially available quercetin supplements are presented in Table 2, highlighting the different formulation technologies and how the chemical forms may influence the absorption characteristics and relative bioavailability of quercetin.

Compared with conventional crystalline quercetin, phytosome formulations appear to offer the most consistent improvements in its bioavailability by forming stable complexes between quercetin and phospholipids, which promote a higher membrane permeability and intestinal absorption. Liposomal formulations also improve the absorption by increasing its solubility and protecting the compound from degradation in the gastrointestinal tract. In contrast, supplements based on standardized plant extracts of Sophora japonica display a much more variable bioavailability, which is influenced by the flavonoid composition of the extract and the interactions between the constituents of the plant matrix. These observations suggest that the formulation technology is one of the determining factors in the biological efficacy of quercetin supplements, sometimes having a greater impact than the administered dose. Furthermore, these facts emphasize that a comparison between commercially available products should extend beyond the declared quercetin content and include its formulation technology, pharmacokinetic performance and quality assurance, all of which may substantially influence the biological efficacy [12].

### 5.3. Regulatory Perspectives and Future Challenges

The rapid expansion of quercetin formulations, particularly those designed to enhance bioavailability, has increased the need for regulatory frameworks capable of considering not only administered dose but also differences in systemic exposure among formulations. Consequently, modern safety evaluations increasingly integrate bioavailability and exposure assessment into the risk characterization of food supplement ingredients.

To date, no internationally harmonized tolerable upper intake level (UL) has been established for quercetin-containing dietary supplements. Regulatory approaches differ between jurisdictions, but safety evaluations generally focus on anticipated exposure, intended conditions of use, available toxicological evidence, and bioavailability rather than on the demonstration of therapeutic efficacy. In the United States, quercetin has been categorized as a Generally Recognized as Safe (GRAS) substance for specific food applications and use levels. Importantly, GRAS status does not constitute therapeutic approval and applies only to the intended conditions of use evaluated by the U.S. Food and Drug Administration (FDA) [85,86].

In the European Union, food supplements are regulated as foods rather than medicinal products and are therefore assessed primarily from a safety perspective rather than an efficacy perspective. Recent guidance issued by the European Food Safety Authority (EFSA) has further emphasized the importance of integrating relative bioavailability into safety assessments, recognizing that systemic exposure may differ substantially between chemical forms and formulations of the same compound [87].

A particularly relevant recent assessment was published by the Norwegian Scientific Committee for Food and Environment (VKM) in 2024. Following the evaluation of available toxicological and human data, the committee concluded that supplementation with 500 mg/day of quercetin dihydrate could be considered safe for healthy adults under the conditions evaluated. However, the report also emphasized that these conclusions should not be automatically extrapolated to vulnerable populations, including pregnant or lactating women, children, or individuals with significant comorbidities. Furthermore, the available evidence was considered insufficient to draw firm conclusions regarding prolonged uncontrolled use or substantially higher exposure levels [88].

The Canadian regulatory framework offers an additional perspective on the safety evaluation of quercetin-containing products. Under the natural health products (NHP) system, products undergo premarket assessment and must demonstrate acceptable safety and quality profiles together with appropriate conditions of use. Regulatory guidance further emphasizes caution in vulnerable populations and consideration of potential interactions between natural health products and conventional medications [89,90].

A further regulatory consideration concerns the substantial difference between dietary exposure and supplemental intake. Habitual dietary intake of quercetin is generally achieved through the consumption of fruits, vegetables, tea, and other plant-derived foods, whereas dietary supplements frequently provide concentrated doses that may substantially exceed those obtainable from a conventional diet. Consequently, safety cannot be inferred solely from the long history of dietary exposure, and supplemental use requires separate evaluation based on the intended dose, duration of use, and expected systemic exposure [17,88].

An important limitation of the current regulatory landscape is the absence of an officially established tolerable upper intake level (UL) for quercetin by major regulatory authorities. Therefore, risk characterization relies primarily on available toxicological evidence, human intervention studies, estimated exposure levels, and formulation-specific bioavailability data rather than on a universally accepted maximum daily intake threshold. This situation reflects the current limitations of the evidence base, particularly regarding long-term supplementation and emerging delivery technologies [87,88].

Advances in formulation technologies have introduced new challenges for the regulatory evaluation of quercetin safety. Modern delivery systems, including phytosomal, liposomal, nanoemulsion-based, and other advanced formulations, may substantially increase systemic exposure compared with conventional quercetin preparations. As a result, two products providing the same nominal dose may not necessarily generate equivalent biological exposure. This consideration is becoming increasingly relevant as regulatory authorities place greater emphasis on bioavailability and exposure assessment when evaluating the safety of food supplement ingredients [87].

Currently, the available evidence supports a favorable safety profile of quercetin within the dose ranges investigated to date, particularly in healthy adults. Nevertheless, important uncertainties remain regarding long-term supplementation, vulnerable populations, cumulative exposure from multiple sources and the safety implications of emerging high-bioavailability formulations. Future regulatory frameworks are therefore expected to increasingly incorporate formulation-specific bioavailability, pharmacokinetic data and real-world exposure assessment to support evidence-based recommendations for safe quercetin use.

## 6. Molecular Mechanisms, Pharmacological Activities and Therapeutic Potential of Quercetin

**Figure 6:**
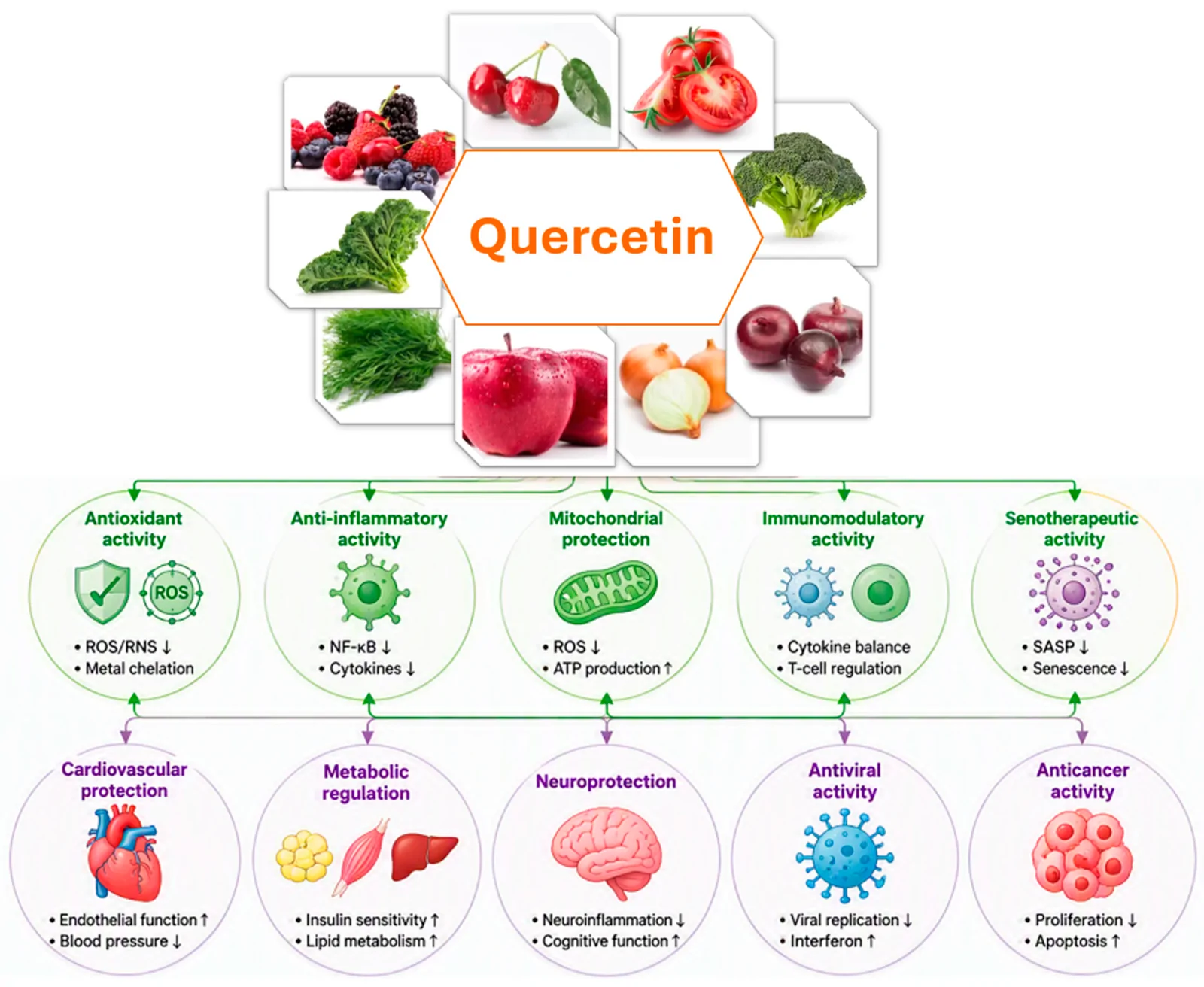
The pharmacological activities and therapeutic potential of quercetin—↑ indicates increased/upregulated activity, whereas ↓ indicates decreased/downregulated activity. Created by the authors based on the data reported in this section.

Quercetin is a pleiotropic bioactive compound capable of modulating interconnected pathways involved in redox homeostasis, inflammation, mitochondrial function, metabolism and cellular stress adaptation [91,92]. Its pharmacological activity is closely related to structural features such as the catechol moiety of the B ring, the multiple hydroxyl substituents and the conjugated C2=C3 double bond, which support redox activity, metal chelation and interactions with intracellular signaling pathways [93,94]. Figure 6 shows its beneficial effects on oxidative stress, inflammation, metabolism, immune regulation, and disease prevention.

Nevertheless, interpretation of these effects requires consideration of pharmacokinetic exposure. Poor oral bioavailability, extensive conjugation, microbial biotransformation and tissue-specific distribution mean that concentrations producing effects in experimental systems may not always be achievable in humans. This limitation is particularly relevant for studies employing supraphysiological concentrations of the aglycone, whereas circulating quercetin is present predominantly as conjugated and microbially derived metabolites [30,95]. The following sections therefore examine not only the proposed molecular mechanisms and pharmacological effects but also the strength and translational relevance of the supporting evidence.

### 6.1. Quercetin as a Modulator of Redox, Inflammatory and Mitochondrial Signaling

The antioxidant activity of quercetin involves both direct chemical effects and regulation of endogenous cellular defenses. Its catechol group, hydroxyl substituents and conjugated structure facilitate electron or hydrogen donation, stabilization of reactive intermediates and chelation of transition metals such as iron and copper. These properties may limit reactive oxygen and nitrogen species, Fenton-type reactions and oxidative damage to lipids, proteins and DNA [33,96,97,98,99,100,101]. However, direct radical scavenging alone is unlikely to explain its in vivo activity given the extensive metabolism and low circulating concentrations of the unconjugated aglycone.

Although quercetin affects multiple interconnected molecular pathways, the strength of evidence differs considerably among the reported pharmacological effects. Antioxidant and anti-inflammatory mechanisms are consistently supported by experimental studies, whereas evidence for disease modification, senolytic activity, antiviral efficacy and anticancer applications remains predominantly preclinical. Therefore, the following subsections distinguish the molecular findings from clinically demonstrated effects and emphasize the principal limitations affecting their translation.

Quercetin also influences cellular redox adaptation through modulation of the Nrf2/Keap1/ARE pathway. Activation of Nrf2-associated signaling has been linked to increased expression of superoxide dismutase, catalase, glutathione peroxidase, heme oxygenase-1 and other cytoprotective systems [96,102,103,104,105,106,107]. These responses may contribute to the preservation of mitochondrial membrane integrity, reduction of mitochondrial ROS generation and maintenance of cellular bioenergetics under oxidative stress [108,109,110,111].

Redox regulation is closely connected with the anti-inflammatory effects of quercetin. In experimental models, quercetin inhibits NF-κB-associated transcription and reduces mediators such as TNF-α, IL-1β, IL-6, cyclooxygenase-2 and inducible nitric oxide synthase. It can also modulate MAPK- and SIRT1-associated pathways and suppress NLRP3 inflammasome activation, thereby interfering with the reciprocal amplification of oxidative stress and inflammation [103,112,113,114,115,116,117].

Collectively, these mechanisms provide a plausible biological basis for the cardiometabolic, neuroprotective, immunomodulatory and anticancer effects discussed below [118,119,120,121]. Nevertheless, the evidence is derived predominantly from cell and animal models, with substantial heterogeneity in dose, formulation and experimental conditions. Human evidence remains more limited, and it is not yet clear whether all molecular effects reported at high experimental concentrations translate into clinically meaningful outcomes after oral administration [122,123].

### 6.2. Quercetin and the Biology of Aging

Cellular senescence has emerged as one of the central biological hallmarks of aging and is increasingly recognized as a common pathogenic mechanism linking numerous chronic diseases, including cardiovascular disease, neurodegeneration, metabolic disorders, musculoskeletal degeneration and cancer. While transient senescence contributes to physiological tissue remodeling and wound healing, persistent accumulation of senescent cells promotes chronic inflammation, impaired tissue regeneration and progressive functional decline. Consequently, senescence has become an important therapeutic target for interventions aimed at extending health span rather than simply increasing lifespan [124,125]. Within the current geroscience framework, targeting cellular senescence is considered a promising strategy for delaying multiple age-related pathologies simultaneously rather than treating each disease independently, reflecting the concept that aging itself represents a modifiable biological process [125].

Cellular senescence is characterized by stable cell-cycle arrest induced by mechanisms including DNA damage, oxidative stress, mitochondrial dysfunction and chronic inflammation. Although senescence contributes to physiological processes such as tissue repair and tumor suppression, persistent accumulation of senescent cells promotes age-associated dysfunction through secretion of pro-inflammatory mediators collectively known as the senescence-associated secretory phenotype (SASP). Because of its ability to modulate pathways involved in oxidative stress, inflammatory signaling and cellular survival, quercetin has emerged as a candidate senotherapeutic compound with predominantly senomorphic and context-dependent senolytic properties. This distinction is particularly important because contemporary senotherapy aims not only to eliminate senescent cells but also to modulate their deleterious secretory phenotype while preserving beneficial physiological functions associated with transient senescence [125,126].

The detrimental effects of senescent cells are largely mediated by the senescence-associated secretory phenotype (SASP), which includes pro-inflammatory cytokines, chemokines, growth factors and matrix-remodeling enzymes. Persistent SASP signaling promotes chronic inflammation, tissue remodeling, paracrine senescence and impaired immune homeostasis. Experimental evidence suggests that quercetin may attenuate SASP-associated signaling through modulation of NF-κB, Nrf2 and PI3K/Akt pathways, supporting a predominantly senomorphic and context-dependent senolytic activity [126,127].

Quercetin has been associated with the regulation of signaling pathways involved in senescent-cell survival and SASP production, including PI3K/Akt/mTOR, AMPK, NF-κB and Nrf2 signaling. Current evidence suggests that its predominant activity may often be senomorphic rather than purely senolytic, since quercetin frequently reduces inflammatory signaling, oxidative stress burden and senescence-associated markers without necessarily inducing complete elimination of senescent cells [103,110]. By attenuating SASP-associated cytokine production and restoring redox homeostasis, quercetin may reduce the propagation of paracrine senescence and chronic tissue inflammation, two processes increasingly recognized as major drivers of age-related functional decline. In experimental models of intervertebral disc degeneration, quercetin reduced senescence markers and SASP-associated mediators through modulation of the Nrf2/NF-κB axis, supporting its context-dependent role in cellular senescence control [128,129].

Cellular senescence contributes to the progression of several chronic disorders, including cardiovascular and metabolic diseases, osteoarthritis, intervertebral disc degeneration, pulmonary fibrosis and neurodegenerative conditions. Although these associations support the therapeutic relevance of targeting senescent cells, the contribution of senescence varies among tissues and disease stages, and the clinical benefits of quercetin-mediated senescence modulation remain insufficiently established [124,125].

Quercetin has also been evaluated in combination with established senotherapeutic agents, particularly dasatinib, highlighting the potential of combination approaches for targeting senescent-cell survival pathways [124,126]. The therapeutic rationale and current evidence supporting these combination strategies are discussed in greater detail in the section on combination therapy.

Beyond individual age-related diseases, quercetin has attracted interest as a potential contributor to healthy aging through attenuation of chronic low-grade inflammation, preservation of mitochondrial function and modulation of oxidative stress-sensitive pathways. However, these effects remain predominantly supported by experimental evidence, and direct demonstration of improved health span or functional independence in humans is currently lacking [130].

Despite growing interest in senotherapeutics, translation toward clinical application remains limited by low oral bioavailability, heterogeneity between experimental models and lack of standardized biomarkers for senescence assessment in humans. Another important challenge is the dual biological role of senescence. While chronic accumulation of senescent cells contributes to aging and age-related diseases, transient senescence remains essential for tissue repair, embryonic development and tumor suppression. Consequently, indiscriminate elimination of senescent cells may not always be desirable, emphasizing the need for selective and context-dependent senotherapeutic strategies.

As such, quercetin should currently be regarded as a promising experimental senotherapeutic agent rather than a clinically validated anti-aging intervention, because its long-term safety, effective human dose and appropriate patient selection criteria remain insufficiently defined [125,126].

### 6.3. Organ-System Effects of Quercetin

#### 6.3.1. Cardiovascular Protection

Quercetin has attracted considerable interest in cardiovascular research because endothelial dysfunction, vascular oxidative stress and chronic low-grade inflammation are key mechanisms underlying the initiation and progression of cardiovascular diseases [131,132]. Rather than acting through a single mechanism, quercetin appears to modulate multiple interconnected pathways involved in vascular homeostasis, redox balance and endothelial function, highlighting its potential cardioprotective role across diverse cardiovascular conditions [133,134].

One of the best-characterized mechanisms involves preservation of endothelial function through maintenance of nitric oxide bioavailability and attenuation of oxidative stress-induced endothelial injury. Experimental studies indicate that quercetin may reduce excessive ROS generation, preserve endothelial nitric oxide synthase (eNOS) activity and improve vascular responsiveness under conditions associated with endothelial dysfunction [131,135]. In addition, quercetin may protect the vascular endothelium by attenuating inflammatory activation, reducing oxidative damage and modulating signaling pathways involved in vascular tone and endothelial homeostasis, mechanisms particularly relevant in atherosclerosis, hypertension and cardiometabolic disease progression [136,137].

Beyond its effects on endothelial function, quercetin has been extensively investigated for its potential role in blood pressure regulation and vascular function. Several studies suggest that quercetin may influence vascular tone through mechanisms involving nitric oxide signaling, endothelial nitric oxide synthase activity and improved vascular relaxation responses, thereby contributing to enhanced vascular responsiveness under pathological conditions [133,138]. Experimental evidence further indicates that quercetin may attenuate vascular dysfunction by reducing oxidative burden, modulating inflammatory signaling and limiting mechanisms associated with vascular stiffness and endothelial injury, processes strongly implicated in hypertension and cardiometabolic disease progression [139,140]. Clinical evidence remains more heterogeneous. Meta-analyses and translational studies suggest that quercetin supplementation may modestly improve selected cardiovascular outcomes, particularly systolic blood pressure and markers of vascular dysfunction. However, these effects appear to depend on dose, baseline cardiometabolic status, formulation and intervention duration [138,141].

Atherosclerosis represents another major cardiovascular context in which quercetin has attracted considerable attention. Oxidative modification of low-density lipoproteins, endothelial activation and persistent inflammatory signaling represent critical events during atherogenesis, and several experimental studies suggest that quercetin may interfere with these mechanisms through antioxidant and anti-inflammatory actions [142]. Experimental evidence further indicates that quercetin may reduce lipid peroxidation, attenuate oxidative modification of circulating lipoproteins and modulate inflammatory pathways involved in vascular injury and plaque progression, thereby influencing multiple stages of atherosclerotic development [143,144]. Additional mechanisms proposed for cardiovascular protection include inhibition of platelet activation and attenuation of vascular inflammation, both of which may contribute to reduced thrombotic risk and improved vascular homeostasis [117,119].

Beyond its vascular effects, quercetin has been investigated in myocardial remodeling, fibrosis and ischemia-related injury. Experimental models suggest that it may attenuate oxidative injury, inflammatory signaling, fibroblast activation and excessive extracellular matrix deposition, thereby limiting adverse cardiac remodeling [134,140,142,145]. However, the strength of evidence differs among cardiovascular outcomes. Modest reductions in systolic blood pressure and improvements in selected vascular markers are supported by some clinical and meta-analytic evidence, whereas the proposed anti-atherosclerotic, anti-fibrotic and myocardial-protective effects remain predominantly preclinical. Interpretation is further complicated by differences in dose, formulation, treatment duration and baseline cardiometabolic status. Consequently, current evidence does not support standardized therapeutic recommendations, and well-designed trials using pharmacokinetically characterized formulations are required to identify clinically relevant doses and patient populations most likely to benefit [123,141].

#### 6.3.2. Metabolic Disorders

Metabolic disorders represent one of the most intensively investigated fields of quercetin research because oxidative stress, chronic low-grade inflammation, mitochondrial dysfunction and impaired metabolic flexibility are central mechanisms linking obesity, insulin resistance, type 2 diabetes mellitus (T2DM) and metabolic-dysfunction-associated steatotic liver disease (MASLD) [146,147]. These pathological processes are highly interconnected, as disturbances in mitochondrial function and redox homeostasis promote altered substrate utilization, excessive lipid accumulation and progressive metabolic deterioration across multiple tissues involved in systemic energy regulation [147,148]. Furthermore, persistent metabolic overload promotes chronic activation of inflammatory pathways, creating a self-perpetuating cycle between oxidative stress, inflammation and impaired metabolic adaptation that accelerates disease progression [149,150].

Quercetin appears to influence interconnected signaling networks involved in nutrient sensing, energy balance and cellular adaptation to metabolic stress [146,149]. Accumulating data suggest that quercetin may regulate AMP-activated protein kinase-associated pathways, modulate SIRT1-related mechanisms and interfere with redox-sensitive signaling cascades involved in mitochondrial homeostasis and metabolic regulation [147,151]. Through these mechanisms, quercetin may influence glucose utilization, lipid handling and mitochondrial adaptation while attenuating inflammatory responses associated with metabolic dysfunction and insulin resistance [5,149]. Consequently, quercetin is increasingly investigated as a pleiotropic metabolic modulator rather than solely as an antioxidant compound, particularly in pathological contexts where oxidative stress, inflammation and metabolic dysregulation coexist and mutually reinforce one another [146,147].

Obesity-associated metabolic dysfunction reflects not only excessive fat accumulation but also adipocyte hypertrophy, altered adipokine secretion, immune-cell infiltration and impaired lipid handling, which collectively promote chronic inflammation and insulin resistance [147,148,149,150]. Experimental studies suggest that quercetin may modulate adipogenesis, attenuate adipocyte hypertrophy and reduce adipose-tissue inflammation [147,152]. It has also been associated with increased expression of thermogenic regulators, including uncoupling protein-1 (UCP1) and peroxisome proliferator-activated receptor gamma coactivator-1α (PGC-1α), suggesting a potential role in white-adipose-tissue browning and energy expenditure. However, these thermogenic and body-weight-related effects are supported predominantly by cellular and animal models and have not been consistently demonstrated in humans. Modulation of inflammatory, adipokine and mitochondrial pathways may nevertheless contribute to improved insulin responsiveness under obesity-associated metabolic stress [146,151,153].

Type 2 diabetes mellitus (T2DM) is characterized by progressive impairment of glucose homeostasis resulting from the interaction between insulin resistance, β-cell dysfunction and chronic metabolic inflammation. Persistent hyperglycemia promotes oxidative stress and mitochondrial dysfunction, processes that further aggravate defects in insulin responsiveness and metabolic regulation [154,155]. Experimental evidence suggests that quercetin may influence glucose metabolism through multiple complementary mechanisms, including enhancement of glucose uptake, modulation of insulin-sensitive signaling pathways and regulation of glucose transporter activity, thereby improving cellular glucose handling and metabolic adaptation [131,156]. In addition, quercetin has been associated with preservation of pancreatic β-cell function through attenuation of oxidative stress, reduction of apoptosis and modulation of intracellular protective pathways, mechanisms that may contribute to improved glycemic regulation under diabetic conditions [157,158]. Maintenance of mitochondrial homeostasis within pancreatic β-cells may further contribute to preservation of insulin secretory capacity under conditions of chronic glucotoxicity and oxidative stress. Overall, current evidence supports the multitarget role of quercetin in glucose homeostasis, although translational applicability remains constrained by bioavailability limitations and insufficient clinical evidence [159,160].

Metabolic dysfunction-associated steatotic liver disease (MASLD) is closely linked to obesity, insulin resistance and disturbances in hepatic lipid metabolism. Excessive hepatic lipid accumulation promotes lipotoxicity, mitochondrial dysfunction and oxidative stress, processes that contribute to inflammatory activation and progression from simple steatosis toward more advanced hepatic injury [141,161]. Because hepatic and systemic metabolic dysfunction are strongly interconnected, impaired liver metabolism may further aggravate insulin resistance and whole-body metabolic homeostasis [162,163]. Available evidence indicates that quercetin may modulate pathways involved in MASLD progression, including hepatic lipid handling, oxidative stress responses and inflammatory signaling. Recent data indicate that quercetin can reduce hepatic lipid accumulation and improve liver-related metabolic and inflammatory markers, although clinical evidence remains limited and heterogeneous. In addition, quercetin has been reported to attenuate endoplasmic reticulum stress and preserve mitochondrial function, thereby limiting progression from simple steatosis toward steatohepatitis and fibrosis in experimental models [161,163].

The strength of evidence differs among metabolic outcomes. Effects on adipose-tissue browning, pancreatic β-cell protection and prevention of MASLD progression are supported mainly by cellular and animal models, whereas human studies have primarily evaluated glycemic, lipid, inflammatory and anthropometric outcomes, with inconsistent results [159,160,161,163]. Direct comparison remains difficult because studies differ in population, dose, intervention duration, chemical form and formulation strategy [14]. Moreover, poor oral bioavailability and interindividual variability prevent consistent systemic exposure, while the doses used in experimental models may not correspond to those achievable through conventional supplementation [48]. Consequently, current evidence supports a plausible adjunctive metabolic role for quercetin but does not establish efficacy as a treatment for obesity, T2DM or MASLD. Future trials should use standardized, pharmacokinetically characterized formulations and distinguish outcomes according to the specific metabolic disorder investigated.

#### 6.3.3. Neuroprotective Effects

Neuroprotective effects represent another major area of interest in quercetin research because oxidative stress, mitochondrial dysfunction, neuroinflammation and progressive neuronal loss are central mechanisms involved in neurodegenerative disorders and age-associated cognitive decline. The central nervous system is particularly vulnerable to oxidative damage due to its elevated metabolic activity, high oxygen consumption and susceptibility to mitochondrial impairment, making disturbances in redox homeostasis especially relevant for neuronal survival and function [91,164]. Persistent activation of neuroinflammatory pathways further contributes to neuronal dysfunction through chronic microglial activation, excessive cytokine production and amplification of oxidative-damage-associated signaling cascades [165,166]. Quercetin appears to influence multiple interconnected pathways involved in neuronal homeostasis, mitochondrial preservation and inflammatory regulation. Experimental studies indicate that quercetin can regulate oxidative stress-sensitive pathways, modulate microglial activation and attenuate neuroinflammatory signaling implicated in neuronal dysfunction and degeneration [167]. Because of these mechanisms, quercetin is increasingly being investigated as a multitarget neuroprotective compound with potential relevance across neurodegenerative and neurovascular disorders, although translation toward clinical application remains constrained by bioavailability limitations and insufficient human evidence.

Persistent microglial activation and dysregulated inflammatory signaling contribute to neuronal injury through excessive cytokine production, NLRP3 inflammasome activation and amplification of oxidative and mitochondrial stress [34,168,169]. Experimental studies suggest that quercetin may modulate microglial polarization, suppress inflammasome-associated signaling and activate Nrf2-dependent antioxidant responses [108,170]. Preservation of mitochondrial function may further reduce ROS-mediated inflammatory amplification and support neuronal survival [91,164]. However, these mechanisms have been demonstrated predominantly in cellular and animal models and cannot be assumed to occur with comparable magnitude at the concentrations achieved in the human brain after oral supplementation.

Alzheimer’s disease (AD) represents one of the most extensively investigated neurodegenerative contexts for quercetin because multiple pathogenic mechanisms implicated in disease progression are potentially influenced by its pleiotropic biological activity. Experimental studies indicate that quercetin may attenuate Aβ-induced neurotoxicity by reducing oxidative damage, modulating inflammatory signaling and interfering with amyloid aggregation pathways [171]. In parallel, quercetin has been associated with attenuation of tau-associated pathology, preservation of synaptic integrity and improvement of learning and memory performance in preclinical models, suggesting broader effects on neuronal homeostasis beyond direct antioxidant activity [170,172]. Recent evidence further suggests that quercetin may preserve synaptic plasticity through modulation of brain-derived neurotrophic factor (BDNF)-associated signaling, thereby supporting neuronal communication, synaptic maintenance and cognitive function in experimental models of AD [91,164]. Modulation of mitochondrial function and regulation of oxidative stress-sensitive pathways may further contribute to neuronal preservation under neurodegenerative conditions [91,173,174].

Parkinson’s disease (PD) is characterized by progressive degeneration of dopaminergic neurons accompanied by mitochondrial dysfunction, oxidative stress and pathological protein aggregation. Loss of dopaminergic signaling within the basal ganglia contributes to motor impairment, while pathological accumulation of α-synuclein further exacerbates neuronal dysfunction and disease progression [175,176]. Experimental studies suggest that quercetin may exert neuroprotective effects in PD models by attenuating oxidative stress, preserving mitochondrial function and regulating pathways associated with neuronal survival. Quercetin has also been associated with reduced neurotoxicity, improved mitochondrial homeostasis and interference with α-synuclein-associated pathogenic mechanisms, suggesting neuroprotective actions that extend beyond direct free-radical scavenging [177]. Preservation of mitochondrial quality control, including maintenance of mitochondrial membrane integrity and regulation of mitophagy-related pathways, has also been proposed as a mechanism contributing to neuronal survival in experimental PD models [177,178]. Collectively, modulation of inflammatory signaling and improvement of cellular stress responses may contribute to preservation of dopaminergic neurons under neurodegenerative conditions [42,178].

Although quercetin has shown neuroprotective activity in models of Alzheimer’s and Parkinson’s diseases, the evidence remains predominantly preclinical. Poor aqueous solubility, extensive metabolism and restricted or incompletely characterized blood–brain barrier penetration limit the extent to which experimental findings can be extrapolated to humans [108,178]. An additional uncertainty concerns whether circulating conjugated metabolites, rather than the aglycone used in many experimental studies, reach neural tissues at biologically effective concentrations. Comparisons between studies are further complicated by heterogeneous models, doses and formulations [174,177]. Nanoformulations and brain-targeted carriers may improve central nervous system delivery, but evidence that these systems produce clinically meaningful neurological benefits remains insufficient. Consequently, quercetin should currently be regarded as a promising neuroprotective candidate rather than an established intervention for neurodegenerative disease [108,178].

### 6.4. Immunomodulatory and Anti-Allergic Effects

Quercetin may influence both innate and adaptive immune responses through modulation of cytokine production, macrophage activation and lymphocyte-associated signaling [178,179,180]. Experimental studies also suggest effects on T-cell regulation and Th1/Th2 polarization, mechanisms potentially relevant to chronic inflammatory and allergic conditions. These actions should be interpreted as context-dependent immunomodulation rather than generalized immune stimulation, because the direction and magnitude of the response depend on the cell type, inflammatory environment, dose and formulation investigated [178].

The anti-allergic activity of quercetin has been associated primarily with inhibition of mast-cell activation and calcium-dependent degranulation, resulting in reduced release of histamine, leukotrienes and other proallergic mediators [179,181]. Experimental models further suggest modulation of IgE-associated responses, Th1/Th2 balance and cytokines involved in eosinophilic inflammation [118,182]. Although these mechanisms support potential activity in allergic disorders, clinical evidence remains limited and does not yet establish the effective dose, formulation or patient population most likely to benefit.

The anti-infective activity of quercetin has been extensively investigated in preclinical models against a broad spectrum of pathogens. Proposed antibacterial mechanisms include the disruption of microbial membrane integrity, interference with biofilm formation and modulation of virulence-associated pathways, mechanisms that may reduce microbial survival and pathogenicity under experimental conditions [183]. In addition, quercetin has demonstrated inhibitory activity against both Gram-positive and Gram-negative microorganisms, although antimicrobial potency appears highly dependent on formulation, concentration and microbial species investigated.

Despite the promising immunomodulatory, antibacterial and anti-allergic properties reported in experimental studies, translation toward clinically relevant applications remains limited. Considerable heterogeneity in formulations, administered doses, treatment duration and investigated populations complicates direct comparison between studies and reduces reproducibility across clinical settings [62]. In addition, many reported antibacterial and anti-allergic effects derive predominantly from preclinical models, whereas human studies remain limited in number, sample size and methodological consistency, preventing firm therapeutic recommendations at present [118,184].

### 6.5. Antiviral Activity

Viral infections remain a major global health challenge because the continuous emergence of novel viral pathogens, viral mutations and antiviral resistance limits the long-term effectiveness of existing therapeutic strategies. Consequently, increasing attention has shifted toward host-directed bioactive compounds capable of simultaneously interfering with viral replication and modulating host defense mechanisms. Owing to its multitarget biological activity, quercetin has emerged as a promising natural compound with broad-spectrum antiviral potential. Rather than acting through a single mechanism, quercetin appears to interfere with multiple stages of the viral life cycle while simultaneously regulating oxidative stress, inflammatory signaling and antiviral immune responses, thereby potentially limiting viral propagation and virus-associated tissue injury under experimental conditions [14,185]. Experimental studies suggest that quercetin may interfere with viral attachment, cellular entry, genome replication and selected host pathways required for viral propagation. Proposed molecular targets include viral attachment proteins, proteases and RNA-dependent RNA polymerases, although the relevance of individual targets differs among viral families [183,185,186]. Activity has been reported against several DNA and RNA viruses, including influenza, herpes simplex, hepatitis and coronaviruses, predominantly in molecular, cellular and animal models [14,151]. However, evidence derived from molecular docking or enzyme inhibition assays should not be interpreted as demonstrating antiviral efficacy in vivo, particularly when the effective experimental concentrations exceed those achievable after oral administration.

Among the investigated viral diseases, SARS-CoV-2 has received particular attention because molecular docking, in vitro experiments and preliminary clinical studies have suggested that quercetin may interact with the selected formulation influences not only the intestinal absorption but also the stability, tissue distribution and systemic exposure.

Nevertheless, docking affinity does not confirm target inhibition in biological systems, and the available clinical studies are heterogeneous in formulation, dose, concomitant treatment and methodological quality. Current evidence is therefore insufficient to support the routine therapeutic use of quercetin for SARS-CoV-2 infection [184,185]. In addition to its direct antiviral properties, quercetin may exert host-directed antiviral effects by modulating innate immune responses and attenuating excessive inflammation associated with viral infections. Experimental evidence indicates that quercetin can suppress activation of NF-κB-dependent inflammatory pathways, reduce production of pro-inflammatory cytokines and regulate oxidative stress-sensitive signaling, thereby contributing to limitation of virus-induced tissue damage while preserving antiviral immune defense [178,185]. In addition, experimental studies suggest that quercetin may promote type I interferon responses and interferon-stimulated genes during the early stages of infection, thereby contributing to the establishment of an antiviral cellular environment [151]. Because severe viral diseases frequently result from dysregulated host inflammatory responses rather than viral replication alone, these immunomodulatory properties may represent an important complementary mechanism underlying the antiviral activity of quercetin.

The antiviral evidence for quercetin is derived mainly from in vitro assays, molecular modeling and animal studies, while human investigations remain limited and methodologically heterogeneous [184,185]. Poor aqueous solubility, extensive phase II metabolism and low systemic exposure further complicate extrapolation from experimental concentrations to clinically achievable doses. Nanoformulations may improve delivery, but enhanced pharmacokinetics alone does not demonstrate antiviral efficacy or safety. Quercetin should therefore be regarded as an experimental antiviral candidate or potential adjunct rather than an established antiviral therapy. Future studies should identify pharmacologically relevant targets, compare active concentrations with human exposure and evaluate standardized formulations in appropriately controlled clinical trials.

### 6.6. Anticancer Activity

Anticancer activity represents one of the most extensively investigated pharmacological areas of quercetin research because multiple hallmarks of cancer are influenced by oxidative stress, dysregulated signaling pathways and altered cellular metabolism. Experimental evidence suggests that quercetin may interfere with several processes involved in tumor initiation and progression, including uncontrolled proliferation, resistance to apoptosis, chronic inflammation and metabolic reprogramming [123,187]. Rather than acting through a single mechanism, quercetin appears to modulate interconnected signaling networks involved in cell survival, oxidative stress responses and cellular adaptation to oncogenic stimuli [188]. Increasing evidence indicates that quercetin may exert anti-neoplastic effects through regulation of oxidative stress-sensitive pathways, modulation of inflammatory mediators and interference with signaling cascades involved in proliferation and survival of malignant cells [189]. Because carcinogenesis involves simultaneous alterations in multiple cellular pathways, quercetin is increasingly investigated as a pleiotropic anti-cancer compound with potential applications both as a standalone adjunctive strategy and as a sensitizing molecule in combination therapies [190].

Quercetin has demonstrated antiproliferative and proapoptotic activity in multiple cancer-cell models. Proposed mechanisms include modulation of cyclins, cyclin-dependent kinases and checkpoint proteins, resulting in cell-cycle arrest, together with regulation of Bcl-2 family proteins, mitochondrial integrity and caspase-dependent apoptosis [123,191,192,193]. Quercetin may also interfere with metabolic and kinase-mediated survival pathways that support malignant-cell adaptation [187]. However, the direction and magnitude of these effects vary according to tumor type, molecular subtype, exposure time and concentration. Moreover, selective toxicity toward malignant rather than normal cells has not been established consistently, particularly at the relatively high concentrations frequently employed in vitro.

Quercetin has also been reported to inhibit migration, invasion and angiogenesis in experimental cancer models. These effects have been associated with modulation of epithelial–mesenchymal transition, matrix metalloproteinases, adhesion proteins and extracellular matrix remodeling [187,189,194]. Additional proposed targets include VEGF-, HIF-1α- and PI3K/Akt-associated pathways involved in vascular support, hypoxic adaptation and metastatic dissemination [188,189,194]. Nevertheless, most evidence derives from simplified cellular or animal models that do not fully reproduce tumor heterogeneity, stromal interactions and metastatic evolution in patients.

The molecule has attracted considerable interest as a potential sensitizing molecule capable of improving responses to conventional anti-cancer therapies. Experimental studies indicate that quercetin may enhance susceptibility of malignant cells to chemotherapy through modulation of oxidative stress responses, interference with survival-associated signaling pathways and attenuation of mechanisms involved in treatment resistance [190,193]. Regulation of drug-resistance-associated pathways and modulation of cellular stress responses may support improved responsiveness to cytotoxic agents while potentially reducing resistance-related adaptive mechanisms observed in tumor cells [193,194]. The therapeutic potential of quercetin as an adjuvant in combination with conventional anticancer agents, together with recent advances in co-delivery systems, is discussed in a separate section dedicated to combination therapy.

Low solubility, extensive metabolism and variability in systemic exposure continue to complicate extrapolation of experimental findings toward clinical settings and may reduce reproducibility of therapeutic responses observed across different models [62,95]. In addition, considerable heterogeneity regarding tumor models, administered doses and formulation strategies complicates direct comparison between studies and limits standardization of therapeutic approaches [88,194]. Tumor heterogeneity, molecular subtype-specific responses and formulation-dependent differences in systemic exposure further complicate direct extrapolation of experimental findings to clinical oncology.

### 6.7. Quercetin in Combination Therapy

The multitarget pharmacological profile of quercetin has stimulated increasing interest in its use as an adjuvant compound capable of enhancing the efficacy of conventional therapeutic approaches. Rather than replacing established treatments, quercetin is increasingly investigated as a complementary agent that may improve therapeutic responses, reduce treatment-related toxicity and overcome resistance mechanisms through simultaneous modulation of multiple signaling pathways. In parallel, advances in nanotechnology and targeted delivery systems have created new opportunities to improve quercetin bioavailability and facilitate its co-administration with conventional drugs, thereby expanding its translational potential beyond that of the native compound [48,190].

Quercetin has been investigated experimentally in combination with doxorubicin, cisplatin, paclitaxel and 5-fluorouracil, as well as with selected targeted and immune-based therapies [33,60,190,194]. Proposed benefits include enhancement of apoptosis, suppression of prosurvival and epithelial–mesenchymal transition pathways, modulation of drug-efflux transporters and attenuation of resistance-associated signaling. Quercetin has also been reported to reduce selected chemotherapy-associated cardiac, renal and neural toxicities in preclinical models [190,194]. However, chemosensitization and protection of normal tissues are not necessarily simultaneous or universally reproducible. Their occurrence depends on tumor type, drug, dose, administration sequence and redox context, while current evidence remains predominantly preclinical.

The combination of dasatinib and quercetin (D + Q) is one of the most extensively investigated senolytic combinations targeting complementary senescent-cell antiapoptotic pathways. Experimental and early clinical studies have reported reductions in selected senescence-associated markers and improvements in some tissue-specific outcomes [124,125]. Nevertheless, responses vary according to tissue, disease, treatment schedule and patient characteristics. Moreover, because quercetin is administered together with dasatinib, the individual contribution of each component cannot always be distinguished. Larger controlled studies are required to establish efficacy, long-term safety and the patient populations most likely to benefit.

The development of advanced delivery systems has further expanded the therapeutic potential of combination strategies involving quercetin. Liposomes, polymeric nanoparticles, lipid-based nanocarriers and multifunctional co-delivery platforms have been designed to improve quercetin solubility, stability and targeted tissue distribution while enabling simultaneous delivery of quercetin with chemotherapeutic agents or other bioactive compounds. These systems may increase intracellular drug accumulation, improve pharmacokinetic performance and reduce systemic toxicity, thereby addressing several of the limitations associated with conventional quercetin formulations. Several of these nanocarriers also enable co-encapsulation of quercetin with chemotherapeutic drugs or other bioactive molecules, providing synchronized delivery and potentially enhancing synergistic therapeutic effects [33,48].

Despite frequent reports of experimental synergy, relatively few quercetin-containing combinations have progressed to controlled clinical evaluation. Potential pharmacodynamic synergy must be distinguished from additive effects, while possible antagonism should also be considered, particularly when antioxidant activity interferes with therapies that depend partly on oxidative damage. Quercetin may additionally modify drug exposure through interactions with metabolic enzymes and transporters, making administration sequence and pharmacokinetic evaluation essential. Translation therefore requires standardized formulations, dose- and sequence-optimization studies, interaction assessment and biomarker-guided selection of appropriate patients [33,48,190]. Until these requirements are met, quercetin-containing combinations should be regarded as experimental strategies rather than validated therapeutic protocols.

To distinguish the mechanistic plausibility from translational evidence, representative experimental and clinical studies evaluating the principal pharmacological effects of quercetin are summarized in Table 3. Particular attention is given to study design, administered dose, evaluated outcomes and the main limitations affecting interpretation and clinical translation.

Overall, the available evidence reveals a substantial imbalance between translational potential and clinical validation. Most pharmacological effects of quercetin are supported predominantly by in vitro and animal studies employing doses or aglycone concentrations that may not be achievable after conventional oral administration. Human evidence remains limited, heterogeneous and strongly influenced by formulation, treatment duration and interindividual variability. Therefore, future studies should prioritize standardized formulations, pharmacokinetic confirmation of systemic and tissue exposure, clinically relevant endpoints and adequately powered randomized trials rather than further descriptive confirmation of molecular mechanisms.

## 7. Challenges, Limitations and Future Perspectives

Despite the rapidly expanding experimental evidence supporting the biological activities of quercetin, several important challenges continue to limit its translation into clinical practice. One of the main limitations remains the considerable discrepancy between the concentrations used in the experimental models and those achievable in humans following oral administration. Poor aqueous solubility limiting intestinal absorption, extensive phase II metabolism, rapid systemic clearance and microbiota-dependent interindividual variability collectively contribute to low and often unpredictable systemic exposure. Furthermore, the substantial heterogeneity among the published studies with respect to quercetin chemical forms, formulation technologies, administered doses, intervention duration and study populations complicates the direct comparison of findings and limits the development of evidence-based therapeutic recommendations [14,30]. Significant progress has been made through the development of advanced delivery systems designed to improve the physicochemical and pharmacokinetic performance of native quercetin. Liposomes, phytosomal formulations, polymeric nanoparticles, nanoemulsions, self-emulsifying drug delivery systems, lipid nanocarriers and cyclodextrin inclusion complexes have demonstrated enhanced solubility, stability and oral bioavailability, whereas multifunctional nanoplatforms have additionally enabled a much more controlled release, targeted tissue delivery and co-administration with conventional therapeutic agents [33,48].

Another important challenge arises from the recognition that quercetin should not be viewed as an isolated bioactive molecule acting independently of the host. Its biological activity is increasingly understood as the result of various complex interactions among formulation-dependent pharmacokinetics, gut microbiota-mediated biotransformation and host metabolic characteristics. Integrating several approaches, such as microbiome profiling, metabolomics, pharmacogenomics and biomarker-guided approaches, would better explain the variability in clinical responses and identify the populations most likely to benefit from quercetin-based interventions. Such systems-level strategies may support the development of personalized nutrition and precision medicine approaches [35,36,92].

Future research should move beyond demonstrating the biological activity of the molecule and focus on establishing its clinical efficacy. Well-designed randomized controlled trials using standardized formulations, validated biomarkers and clinically relevant endpoints are needed to define optimal dosing strategies, therapeutic indications and long-term safety. Moreover, the integration of artificial intelligence, multiomics technologies and predictive computational models may accelerate formulation optimization, patient stratification and individualized intervention design. Collectively, these advances could facilitate the transition of quercetin from a promising multifunctional phytochemical to an evidence-based component of precision nutrition, nutraceutical interventions and adjunctive therapeutic strategies for chronic diseases.

## 8. Conclusions

Quercetin is one of the most studied dietary flavonoids due to its wide distribution in the plant kingdom and its broad spectrum of biological and pharmacological activities. Current evidence indicates that quercetin acts as a pleiotropic bioactive molecule, capable of modulating multiple signaling pathways involved in the regulation of oxidative stress, inflammation, mitochondrial function, immune response, metabolic homeostasis, and processes associated with cellular aging. Through these complex and interconnected mechanisms, quercetin has attracted considerable interest as a potential nutraceutical and therapeutic agent in the prevention and management of cardiovascular, metabolic, neurodegenerative, infectious, and neoplastic diseases. In recent years, the understanding of the pharmacology of quercetin has expanded significantly beyond its classical role as an antioxidant. Recent research has highlighted the importance of quercetin’s interactions with the gut microbiota, the metabolites generated by microbial biotransformation, the mechanisms involved in cellular senescence, quercetin’s antiviral effects and its use in combination therapies. In parallel, the development of modern delivery systems, including phytosomal, liposomal and nanoencapsulated formulations, has offered new opportunities to improve the stability, absorption and biological performance of quercetin, contributing to overcoming important pharmacokinetic limitations. However, translating experimental results into validated clinical applications remains a challenge. Variability of formulations, differences in bioavailability, heterogeneity of clinical trials and the complexity of the relationship between systemic exposure and biological effects make it difficult to establish standardized therapeutic recommendations. Consequently, high-quality clinical trials, specific pharmacokinetic investigations of different formulations and the identification of relevant biomarkers are needed to allow the correlation of molecular mechanisms with observed clinical benefits. Quercetin can be considered a multifunctional bioactive molecule at the interface of nutrition, pharmacology, and personalized medicine. The future integration of the advances in formulation science, microbiome research, systems biology, and clinical studies will be essential to fully understand quercetin’s potential and develop evidence-based strategies for its safe and effective use in nutraceutical and therapeutic applications.

## Figures and Tables

**Table 1:** Comparative physicochemical characteristics, advantages, limitations and potential applications of quercetin-loaded delivery systems.

Method	Matrix Composition	Technique	Size	Zeta Potential (mV)	Stability	Encapsulation Efficiency (EE%)	Advantages	Main Limitations	Use	References
Nanocochleates	Soya lecithin + cholesterol + CaCl_2_	Calcium- induced cochelation (trapping method)	205.6 nm	−4.3	Improved storage stability and sustained drug release	76.36	Improved oral bioavailability, prolonged release, enhanced cytotoxic activity	Limited colloidal stability and lack of clinical validation	Oral/pharmaceutical delivery	[51]
Chitosan nanoparticles	Chitosan crosslinked with quercetin	Ionic gelation	229.2 nm	+23.6	Stable for at least 7 days without significant particle size variation	79.60	Improved nasal absorption, sustained release, enhanced anti-inflammatory activity	Aggregation tendency and limited stability assessment	Nasal drug delivery	[52]
Pickering emulsion gel	Rice bran cellulose nanocrystals + gelatin	Pickering emulsion gelation	6.91 μm	−11.5	Stable during refrigerated storage (14 days); improved oxidative stability	94.57	Improved antioxidant activity and bioaccessibility	Micrometer-scale particle size and storage-dependent stability	Functional foods/nutraceutical delivery	[23]
Liposomes	Soy phosphatidylcholine (SPC) + cholesterol	Thin-film hydration	30 nm	−20.04 to −18.5	Improved physicochemical stability	42	Enhanced antioxidant activity and sustained release	Moderate encapsulation efficiency and complex preparation procedure	Nutraceutical/pharmaceutical delivery	[53]
Natural oil-based nanostructured lipid carriers (NLCs)	Solid lipid + natural plant oils	Melt-emulsification/ultrasonication	154–182 nm	−40	Good colloidal stability during storage	90.27–99.85	Improved skin retention, antioxidant protection and topical delivery	Dependence on oil composition and restricted applicability to topical delivery	Topical delivery	[54]
Liposomes	Phospholipon^®^ 90 NG	Proliposomal encapsulation/thin-film hydration	577–597 nm	−48	Stable for 6 months at 4 °C	67–77	Markedly improved quercetin stability against degradation	Large particle size and requirement for refrigerated storage	Nutraceutical/pharmaceutical delivery	[28]
Nanoliposomes	Soybean lecithin + cholesterol	Thin-film hydration (evaporation–hydration method)	231.6 nm	−37.5	Good storage stability and sustained intestinal release	63.73	Improved stability, sustained release, high biocompatibility	Formulation-dependent stability characteristics	Oral/nutraceutical delivery	[34]
Liposomes	Dimyristoyl phosphatidylglycerol (DMPG) + cholesterol	Thin-film hydration	188.5–253.5 nm	−40.33	Good physical stability; precursor for nanocochleate formulation	68.7–79.4	Improved encapsulation and sustained release	Large particle size and complex manufacturing procedure	Oral/pharmaceutical delivery	[55]

**Table 2:** Representative commercially available quercetin supplements according to formulation technology.

Formulation Technology	Mechanism of Enhanced Absorption	Relative Bioavailability	Representative Products (Dose)
Phytosome (quercetin–phospholipid complex)	Complexation with phospholipids, improved transmembrane absorption; the phospholipid shell facilitates membrane permeation and lymphatic uptake	Substantially higher vs. the standard crystalline quercetin	Quercetin Phytosome (250 mg) [63] Bio-Quercetin (30 mg) [64] Fast-C^®^ and Bio-Quercetin Phytosome (15 mg) [65]
Liposomal encapsulation	Encapsulation in the lipid vesicles protects quercetin from degradation and improves its aqueous solubility	Moderately to substantially higher vs. the standard crystalline quercetin	Quercetin Plus (100 mg) [66] Quercetin Capsules (250 mg) [67] Quercetin liquid (250 mg/2 tsp) [68] Liposomal Quercetin Shield (80 mg) [69]
Standard crystalline quercetin (quercetin dihydrate)	Passive diffusion, limited by low aqueous solubility	Baseline (reference form)	Quercetin with Bromelain (800 mg) [70] Quercetin Complex with Ester-C^®^ Plus (500 mg) [71] Quercetin Bromelain (500 mg) [72] Quercetin & Bromelain (250 mg) [73] Quercetin 500 mg (500 mg) [74] Quercetin Bromelain Vitamin C (250 mg) [75] Quercetină Immune Complex (250 mg) [76] Quercetin 500 mg Plus Bromelain 50 mg (500 mg) [77] Quercetin Complex+ (300 mg) [78]
Plant-extract complex (*Sophora japonica* flower bud extract)	Matrix effect and the coexistence of other biologically active compounds, such as flavonoids; co-delivery; absorption profile influenced by the extract composition	Variable, extract-dependent	Quercetin & B5 Complex (150 mg) [79] Quercetin 500 (500 mg) [80] Quercetin 600 mg + Vitamin C + Bioflavonoids (600 mg) [81] Natural Quercetin 500 mg (500 mg) [82] Daily-Quercetin 500 mg (500 mg) [83] Quercetin 98 Complex (260 mg) [84]

**Table 3:** Representative experimental and clinical evidence for the pharmacological effects of quercetin.

Pharmacological Area	Study Design/Model	Quercetin Form and Dose	Methods/ Evaluated Outcomes	Main Findings	Main Limitations	Reference
Cardiovascular and vascular senescence	Randomized placebo-controlled perioperative study in 97 patients with symptomatic coronary artery disease undergoing coronary artery bypass grafting	Quercetin, 500 mg twice daily, from 2 days before surgery until hospital discharge	Vascular reactivity, endothelial function, inflammatory and cellular senescence markers	Quercetin reduced selected vascular senescence and inflammatory markers and improved vascular responses in male patients, whereas comparable benefits were not demonstrated in women	Short intervention; perioperative setting; sex-dependent findings; no assessment of long-term cardiovascular outcomes	[117]
Anti-allergic activity	Systematic review and meta-analysis of 13 preclinical murine studies involving 183 animals	Different quercetin doses and administration protocols	Serum IgE, ovalbumin-specific IgE, histamine, inflammatory cytokines and immune-cell infiltration	Quercetin reduced several biochemical and inflammatory markers associated with allergic responses	Evidence restricted to animal models; substantial heterogeneity in dose, model and outcomes; high statistical heterogeneity for several endpoints	[118]
Cellular senescence	In vitro study using young and senescent human vascular smooth muscle cells	Dasatinib–quercetin combination; single and repeated treatment protocols	Nuclear morphology, chromatin organization and DNA-texture parameters evaluated using fluorescence microscopy and image analysis	Treatment produced partial chromatin changes consistent with rejuvenation in senescent cells but also induced senescence-associated alterations in young cells	In vitro model; combined treatment prevents attribution of effects to quercetin alone; chromatin changes do not demonstrate clinical rejuvenation or safety	[124]
Metabolic effects	Randomized controlled study in 100 patients with type 2 diabetes mellitus	Quercetin, 500 mg/day for 12 weeks, followed by an 8-week washout and a second 12-week intervention	HbA1c, blood pressure, respiratory function, sleep, anxiety and quality-of-life parameters	Supplementation improved HbA1c and selected cardiovascular and patient-reported outcomes	Modest sample size; standard-care control without placebo; multiple outcomes; formulation-dependent exposure was not characterized	[159]
Neuroprotective activity	Experimental Alzheimer’s disease model in Wistar rats	Quercetin, 25 mg/kg/day by oral gavage for 1 month	Behavioral memory tests, amyloid precursor protein expression and brain pro-inflammatory cytokines	Quercetin improved cognitive performance and reduced amyloid-related and inflammatory markers	Animal model; relatively high body-weight-adjusted dose; circulating metabolites and brain exposure were not fully characterized; absence of human confirmation	[172]
Antiviral activity	Systematic review of randomized controlled trials investigating quercetin in COVID-19	Heterogeneous quercetin products, doses and treatment durations	Clinical symptoms, inflammatory markers, disease progression, hospitalization and viral clearance	Several studies reported potential improvements in clinical or inflammatory outcomes	Considerable variation in formulations, doses, concomitant treatments and methodological quality; limited comparability and insufficient evidence for routine clinical use	[184]
Anticancer activity	In vitro study using YD10B and YD38 oral squamous cell carcinoma cells	Quercetin, primarily 50 µM in YD10B and 100 µM in YD38 cells for 24 h	Cell viability, cell-cycle distribution, Annexin V/propidium iodide staining and protein expression analysis	Quercetin induced G1 cell-cycle arrest and apoptosis, with responses influenced by the molecular characteristics of the cell lines	In vitro evidence; concentrations may exceed clinically achievable free-quercetin exposure; only two cell lines; no pharmacokinetic or in vivo validation	[192]
Combination anticancer therapy	In vitro study using T47D breast cancer stem-like cells	Quercetin, 25–100 µM, and doxorubicin, 100–1000 nM, alone or in combination	Cell viability, cell-cycle progression, apoptosis and expression of apoptosis-related proteins	Quercetin promoted cell-cycle arrest and apoptosis and increased selected cytotoxic effects of doxorubicin	In vitro model; high quercetin concentrations; absence of normal-cell and in vivo comparisons; pharmacokinetic interactions and systemic toxicity were not evaluated	[192]

Abbreviations: HbA1c, glycated hemoglobin; IgE, immunoglobulin E.

## Data Availability

No new data were created or analyzed in this study. Data sharing is not applicable.
